# Supplementary material for: Giant charge-to-spin conversion in ferromagnet via spin-orbit coupling
Source: Nat Commun. 2021 Oct 29;12:6254. doi: 10.1038/s41467-021-26445-y (PMC8556288; doi:10.1038/s41467-021-26445-y)
Supplement: Supplementary file 1 — Supplementary information [file 41467_2021_26445_MOESM1_ESM.pdf]

Supplementary Information

Giant charge-to-spin conversion in ferromagnet via spin-orbit coupling

Y. Hibino*, T. Taniguchi, K. Yakushiji, A. Fukushima, H. Kubota, and S. Yuasa

National Institute of Advanced Industrial Science and Technology (AIST), Research Center for Emerging Computing Technologies, Tsukuba, Ibaraki, 305-8568, Japan

**e-mail:* y-hibino@aist.go.jp

Table of contents

Supplementary Note 1: Magnetic Properties and EDX mapping of tri-layer structure
Supplementary Note 2: Magnetic properties of Co/Ni multilayers
Supplementary Note 3: Magnetoresistance curve and MR ratio
Supplementary Note 4: Theoretical analysis of ST-FMR spectra
Supplementary Note 5: Theoretical formula of spin current conductivity
Supplementary Note 6: Magnetic-dependent Charge-to-spin conversion under $+M_{\mathrm{PML}}$ and $-M_{\mathrm{PML}}$ state
Supplementary Note 7: Charge-to-spin conversion in other PML material systems
Supplementary Note 8: Spin-to-Charge conversion in Co/Ni multilayer
Supplementary Note 9: SOT-induced field-free switching using in-plane magnetized ferromagnet source
Supplementary Figure 1: Ferromagnetic resonance properties of tri-layer structure
Supplementary Figure 2: HAADF-STEM image and EDX elemental mapping
Supplementary Figure 3: EDX line profiles of Series C samples
Supplementary Figure 4: Magnetization curves of tri-layer system
Supplementary Figure 5: Magnetoresistance curves of tri-layer system
Supplementary Figure 6: Schematic for theoretical model
Supplementary Figure 7: Detail fitting result of thickness dependence of ξ_{MD} and ξ_{MI}
Supplementary Figure 8: Charge-to-spin conversion under two magnetic states
Supplementary Figure 9: Charge-to-spin conversion in Co/Pt multilayer spin source
Supplementary Figure 10: Spin-pumping induced spin-to-charge conversion in tri-layer structure.
Supplementary Figure 11: Demonstration of SOT-induced field-free switching

Supplementary Note 1:
Magnetic properties and EDX mapping of tri-layer structure

Supplementary Figures 1(a) and 1(b) show the relations between the resonance frequency, external magnetic field, and resonance linewidth of Ferromagnetic resonance (FMR) spectra. The data shown here are on Series A sample with thickness of PML 1.2 nm. The demagnetization field of IML (H_{demag}) and the Gilbert damping constant (α) were evaluated by analyzing these data with the Kittel formula and Eq. 4 in the main text, respectively. Supplementary figures 1(c) and 1(d) show the PML thickness dependences of the H_{demag} and α in Series A samples. H_{demag} shows basically constant value (~116 mT in Series A samples) indicating no correlation with PML thickness. This tendency was also observed in Series B and C samples. In the case of α, its magnitude is slightly enhanced by ~0.002 with increasing the Fe-B thickness. In addition, obtained value of α is larger than intrinsic damping constant of Fe-B previously reported [1]. These findings indicate the presence of spin dissipation in PML via spin pumping [2].

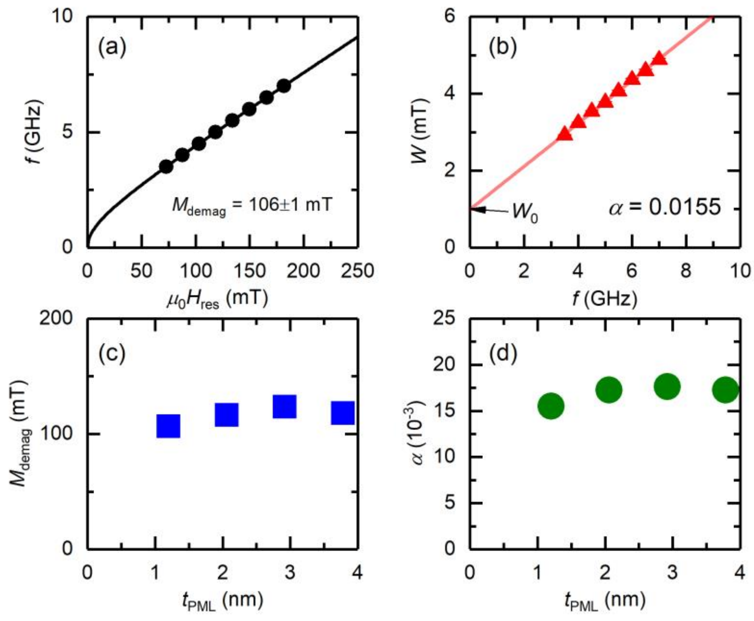

Supplementary Figure 1: Ferromagnetic resonance properties of tri-layer structure. a Resonance frequency f as a function of resonance field H_{res}. Solid curve shows the Kittel formula. **b** Resonance linewidth W as a function of f. Solid line shows the linear fitting result. **c** and **d** PML thickness dependences of **c** effective demagnetization field H_{demag} and **d** Gilbert damping constant in Series A samples**.**

In Supplementary figure 2, we show the results of EDX elemental mapping to check the interface of our tri-layer structure. Here, we measured two Series C samples with Ni concentration x = 0% and x = 69% near the interface. From the EDX mapping, we can find two features: (1) existence of relatively sharp interface between Cu and PML (Co/Ni multilayers), and (2) the Ni element in x = 69% sample concentrates near the interface with Cu, compared to x = 0% sample. The latter is also confirmed from the EDX line profile shown in Supplementary figure 3 where the Ni concentration in Co/Ni multilayers (open circles) near the Cu interface is significantly enhanced in x = 69% sample. These results show that controlling sub-nm-thick layer near the interface is an effective way to manipulate the NM/FM interface.

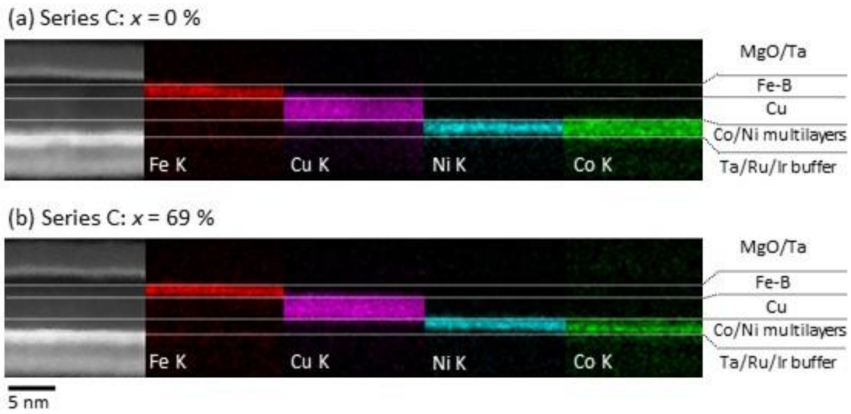

Supplementary figure 2: HAADF-STEM image and EDX elemental mapping. a Series C sample with x = 0% (Co interface) and **b** Series C sample with x = 69% ($Co_{31}Ni_{69}$ interface)**.**

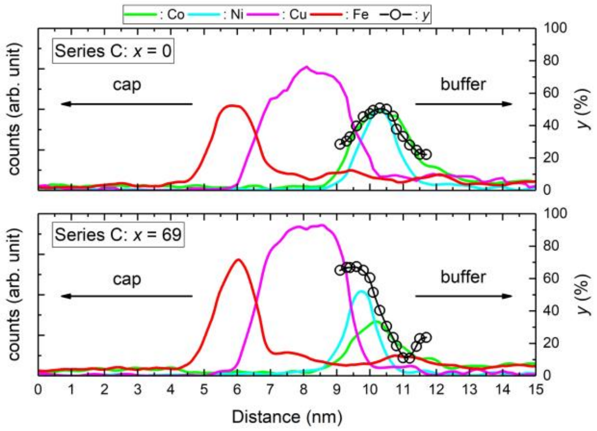

Supplementary figure 3: EDX line profiles of Series C samples. (upper panel) x = 0% (co interface) and (lower panel) x = 69% ($Co_{31}Ni_{69}$ interface). The Ni concentration in the Co/Ni multilayer region y is plotted by the open circles.

Supplementary Note 2:
Magnetic properties of Co/Ni multilayers

The magnetization curves of tri-layer structure under out-of-plane and in-plane magnetic fields ($H_\perp$ and $H_\parallel$) are shown in Supplementary figure 4a (Series A with t_{PML} = 1.2 nm). The sharp magnetization switching near zero field and gradual slope below the saturation field indicate that the present system consists of in-plane magnetized layer and perpendicularly magnetized layer. From the figure, in-plane saturation field of the Co/Ni multilayer ($H_{\mathrm{K}}^{\mathrm{PML}}$) is determined to be 0.8 T (shown as blue arrow). To see whether there is interlayer exchange coupling between the ferromagnetic layers, we prepared reference samples without Fe-B layer. Supplementary figure 4b shows the magnetization curves of the reference sample with t_{PML} = 1.2 nm. Supplementary figure 4c summarizes the $H_{\mathrm{K}}^{\mathrm{PML}}$ of Series A and reference samples. Both samples show relatively high $H_{\mathrm{K}}^{\mathrm{PML}}$ (>0.5 T). In addition, there are basically no differences in $H_{\mathrm{K}}^{\mathrm{PML}}$ between the two systems, indicating negligible interlayer exchange coupling in the tri-layer system.

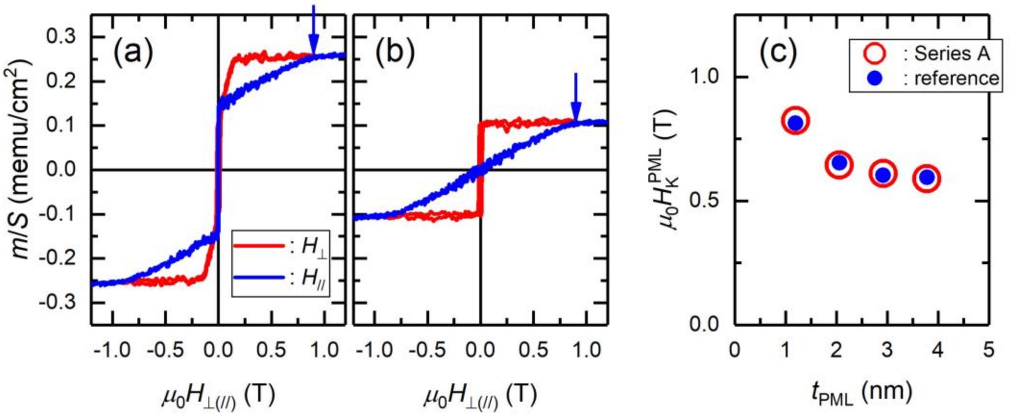

Supplementary figure 4: Magnetization curves of tri-layer system. Magnetization curves of (a) tri-layer structure and (b) reference sample without Fe-B layer. Tri-layer structure corresponds to Series A sample with t_{PML} = 1.2 nm. Blue arrow shows the in-plane saturation field of Co/Ni multilayer. (c) PML thickness dependence of $H_{\mathrm{K}}^{\mathrm{PML}}$ in Series A (open red symbol) and reference samples (solid blue symbols).

Supplementary Note 3:
Magnetoresistance curve and MR ratio

In Supplementary figure 5a, we show the 2-wire resistance R under out-of-plane magnetic field $H_{\perp}$ in Series A sample with t_{PML} = 1.2 nm. The resistance monotonically decreases and settles near 120 mT with increasing $H_{\perp}$. This behavior is explained by the giant magnetoresistance (GMR) effect, where the magnetization IML tilts from in-plane to out-of-plane direction with increasing field. The abrupt drops of resistance near ±50 mT correspond to the magnetization reversal of PML. We converted this magnetoresistance curve into normalized magnetization curve of IML (inset of Supplementary figure 5a) [3] and confirmed that the effective demagnetization field is consistent with that obtained by ST-FMR measurement. In Supplementary figure 5b and 5c, we show the GMR properties of each samples. Because the IML and PML are magnetized orthogonal to each other at zero field, we can obtain the half value of the GMR ratio from the magnetoresistance curve. Series A and B samples (Supplementary figure 5b) show half GMR ratio of 0.7 % in each thickness sample. In the case of Series C samples (Supplementary figure 5c), the GMR ratio monotonically decreases with increasing the Ni concentration at the interface with Cu. This dependence is caused by decrease of spin-dependent scattering and carrier spin polarization with Ni concentration.

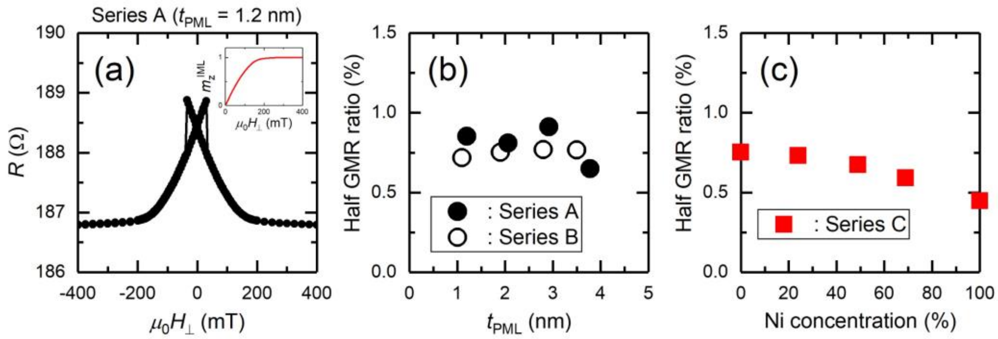

Supplementary figure 5: Magnetoresistance curves of tri-layer system. a Magnetoresistance curve under out-of-plane magnetic field. Inset figure shows the normalized magnetization curve of IML obtained from the magnetoresistance curve. **b** Half GMR ratio of Series A (solid symbols) and B (open symbols) samples. **c** Half GMR ratio of Series C samples.

Supplementary Note 4:
Theoretical analysis of ST-FMR spectra

In this note, we show the derivation of the theoretical formula of spin-torque FMR spectrum.

Section 4.1: Introduction of Landau-Lifshitz-Gilbert (LLG) equation

We assume that the magnetization dynamics in the Fe-B free layer is well described by the Landau-Lifshitz-Gilbert (LLG) equation given by

$$\frac{\mathrm{d}\mathbf{m}_2}{\mathrm{d}t} = -\gamma \mathbf{m}_2 \times \mathbf{H} - \gamma a_J \mathbf{m}_2 \times (\boldsymbol{\sigma} \times \mathbf{m}_2) - \gamma b_J \mathbf{m}_2 \times \boldsymbol{\sigma} + \alpha \mathbf{m}_2 \times \frac{\mathrm{d}\mathbf{m}_2}{\mathrm{d}t}, \qquad (1)$$

where $\mathbf{m}_2$ is the unit vector pointing in the direction of the magnetization in the free layer. The strength of the damping-like torque is denoted as a_J, whereas b_J represents the torque strength consisting of field-like torque and torque due to Oersted field. Note that both a_J and b_J are proportional to the current density. In the following, we add subscripts “dc” and “ac” to a_J and b_J when it is necessary to distinguish a_J and b_J originated from the direct and alternating currents. The gyromagnetic ratio and the Gilbert damping constant are denoted as γ and α, respectively. The unit vector $\boldsymbol{\sigma}$ determines the torque direction. Note that $\boldsymbol{\sigma} = \mathbf{e}_y$ for Oersted field, where $\mathbf{e}_k$ ($k = x, y, z$) denotes the unit vector along the k-direction. On the other hand, $\boldsymbol{\sigma}$ corresponding to spin torque points to either x or y direction, depending on the origin of spin current. For example, $\boldsymbol{\sigma} = \mathbf{e}_y$ for spin currents generated by the spin Hall effect in CoNi and/or spin-filtering effect at CoNi/Cu interface, whereas $\boldsymbol{\sigma} = \mathbf{m}_1 \times \mathbf{e}_y$ is parallel to the x axis for spin current generated by the spin-precession effect at CoNi/Cu interface, where the unit vector $\mathbf{m}_1$ pointing in the magnetization direction of CoNi reference layer is parallel to the z axis in our experiments. For a while, we use the symbol $\boldsymbol{\sigma}$, for generality. The magnetic field consists of an external magnetic field applied in the film-plane and the demagnetization field in the perpendicular direction as

$$\mathbf{H} = \begin{pmatrix} H_{\mathrm{ext}} \cos \varphi_H \\ H_{\mathrm{ext}} \sin \varphi_H \\ -4\pi M m_{2z} \end{pmatrix}, \qquad (2)$$

where φ_H determines the direction of the external magnetic field.

Section 4.2: Determination of oscillation axis

In spin-torque FMR experiment, a small-amplitude oscillation of the magnetization is excited by torques due to spin current and Oersted field generated by alternating current, whereas the torques due to the direct current determine the oscillation axis. Let us here study the theoretical conditions determining the oscillation axis. In terms of zenith and azimuth angles (θ, φ) defined as $\mathbf{m}_2 = (\sin\theta\cos\varphi, \sin\theta\sin\varphi, \cos\theta)$, the LLG equation in the presence of direct current is rewritten as

$$\frac{\mathrm{d}\theta}{\mathrm{d}t} = -\frac{\gamma}{M \sin\theta}\frac{\partial E_{\mathrm{eff}}}{\partial \varphi} - \gamma a_{J(\mathrm{dc})}\frac{\partial}{\partial \theta}\mathbf{m}_2 \cdot \boldsymbol{\sigma} - \alpha \sin\theta \frac{\mathrm{d}\varphi}{\mathrm{d}t}, \tag{3}$$

$$\sin\theta \frac{\mathrm{d}\varphi}{\mathrm{d}t} = -\frac{\gamma}{M}\frac{\partial E_{\mathrm{eff}}}{\partial \theta} - \frac{\gamma a_{J(\mathrm{dc})}}{\sin\theta}\frac{\partial}{\partial \varphi}\mathbf{m}_2 \cdot \boldsymbol{\sigma} + \alpha \frac{\mathrm{d}\theta}{\mathrm{d}t}, \tag{4}$$

where we introduce an effective potential,

$$E_{\mathrm{eff}} = -M \int \mathbf{H} \cdot \mathrm{d}\mathbf{m}_2 - M b_{J(\mathrm{dc})} \mathbf{m}_2 \cdot \boldsymbol{\sigma}. \tag{5}$$

Using supplementary eq. (3) and (4), the steady state solutions of (θ, φ) satisfying $\mathrm{d}\theta/\mathrm{d}t = 0$ and $\mathrm{d}\varphi/\mathrm{d}t = 0$ are determined by the following equations;

$$\begin{aligned} H_{\mathrm{ext}} \sin(\varphi - \varphi_H) - a_{J(\mathrm{dc})}[\sin\theta_\sigma \cos\theta \cos(\varphi - \varphi_\sigma) - \cos\theta_\sigma \sin\theta] \\ - b_{J(\mathrm{dc})} \sin\theta_\sigma \sin(\varphi - \varphi_\sigma) = 0, \end{aligned} \tag{6}$$

$$\begin{aligned} -H_{\mathrm{ext}} \cos\theta \cos(\varphi - \varphi_H) - 4\pi M \sin\theta \cos\theta + a_{J(dc)}\, sin\,\theta_\sigma\, sin(\varphi - \varphi_\sigma) \\ - b_{J(dc)}[\sin\theta_\sigma \cos\theta \cos(\varphi - \varphi_\sigma) - \cos\theta_\sigma \sin\theta] = 0, \end{aligned} \tag{7}$$

where we define θ_σ and φ_σ as

$$\boldsymbol{\sigma} = \begin{pmatrix} \sin\theta_\sigma \cos\varphi_\sigma \\ \sin\theta_\sigma \sin\varphi_\sigma \\ \cos\theta_\sigma \end{pmatrix}. \tag{8}$$

Here, it is useful to note that the magnitude $a_{J(\mathrm{dc})}$ and $b_{J(\mathrm{dc})}$ in spin-torque FMR experiment are on the order of $\alpha|\mathbf{H}|$ because we are interested in the competition between the damping torque and the spin torque. Since the damping constant α is sufficiently small, it is good approximation to neglect terms related to $a_{J(\mathrm{dc})}$ and $b_{J(\mathrm{dc})}$ from supplementary eq. (6) and (7). Then, the oscillation axis becomes parallel to the direction of the in-plane external magnetic field as

$$\theta \cong \frac{\pi}{2}, \quad \varphi \cong \varphi_H. \tag{9}$$

In the following, the symbols (θ, φ) represents the equilibrium direction.

Section 4.3: Introduction of rotating frame

Here, let us introduce a new coordinate XYZ, where Z axis is parallel to the oscillation axis. The transformation from xyz to XYZ coordinate is described by the rotating matrices,

$$\mathcal{R}_y = \begin{pmatrix} \cos\theta & 0 & -\sin\theta \\ 0 & 1 & 0 \\ \sin\theta & 0 & \cos\theta \end{pmatrix}, \quad \mathcal{R}_z = \begin{pmatrix} \cos\varphi & \sin\varphi & 0 \\ -\sin\varphi & \cos\varphi & 0 \\ 0 & 0 & 1 \end{pmatrix}. \tag{10}$$

For example, the magnetization component in the XYZ coordinate is obtained by that in the xyz coordinate as $\mathcal{R}_y \mathcal{R}_z \mathbf{m}_{2xyz} = \mathbf{m}_{2XYZ}$. Similar to this example, we use subscripts xyz and XYZ to distinguish quantities in each coordinate. For example, the polarization vector $\boldsymbol{\sigma}$ and the magnetic field $\mathbf{H}$ in the XYZ coordinate are, respectively, given by

$$\boldsymbol{\sigma} = \begin{pmatrix} \cos\theta \sin\theta_\sigma \cos(\varphi - \varphi_\sigma) - \sin\theta \cos\theta_\sigma \\ -\sin\theta_\sigma \sin(\varphi - \varphi_\sigma) \\ \sin\theta \sin\theta_\sigma \cos(\varphi - \varphi_\sigma) + \cos\theta \cos\theta_\sigma \end{pmatrix}_{XYZ} \equiv \begin{pmatrix} \sigma_X \\ \sigma_Y \\ \sigma_Z \end{pmatrix}. \tag{11}$$

$$\mathbf{H}_{\text{eff}} \equiv \mathbf{H} + a_{J(\text{dc})}\boldsymbol{\sigma} \times \mathbf{m}_2 + b_{J(\text{dc})}\boldsymbol{\sigma} = \begin{pmatrix} H_{XX} m_{2X} - a_{J(\text{dc})}\sigma_Z m_{2Y} \\ a_{J(\text{dc})}\sigma_Z m_{2X} \\ H_{ZX} m_{2X} + H_{ZZ} + b_{J(\text{dc})}\sigma_Z \end{pmatrix}_{XYZ}. \tag{12}$$

Here, we introduce $H_{XX} = -4\pi M \sin^2\theta$, $H_{ZX} = 4\pi M \sin\theta \cos\theta$, and $H_{ZZ} = H_{\text{ext}} \sin\theta \cos(\varphi - \varphi_H) - 4\pi M \cos^2\theta$. Using the approximation given by supplementary eq. (9), these components become $H_{XX} \cong -4\pi M$, $H_{ZX} \cong 0$, and $H_{ZZ} \cong H_{\text{ext}}$. For the latter discussion, we also introduce $H_X = H_{ZZ} - H_{XX} \cong H_{\text{ext}} + 4\pi M$ and $H_Y = H_{ZZ} \cong H_{\text{ext}}$.

Section 4.4: Linearization of the LLG equation

Now we add spin torque due to alternating current to the LLG equation. We describe time-dependences of damping-like and field-like torques as $a_{J(\text{ac})} = \tilde{a}_{J(\text{ac})} e^{i\omega t}$ and $b_{J(\text{ac})} = \tilde{b}_{J(\text{ac})} e^{i\omega t}$, where $\tilde{a}_{J(\text{ac})}$ and $\tilde{b}_{J(\text{ac})}$ are the magnitudes of the damping-like and field-like torques due to the alternating current, whereas $\omega = 2\pi f$ is the angular frequency of the alternating current. Since we are interested in linear response of the magnetization to the alternating current, we also denote the magnetization component in the XYZ coordinate as $m_{2X} = \tilde{m}_{2X} e^{i\omega t}$ and $m_{2Y} = \tilde{m}_{2Y} e^{i\omega t}$, where $|\tilde{m}_{2X,Y}| \ll 1$ in FMR state. Substituting these expressions to supplementary eq. (1), the LLG equation for m_X and m_Y up to their first orders become

$$\begin{pmatrix} i\omega/\gamma - a_{J(\text{dc})}\sigma_Z + \alpha H_X & H_Y + b_{J(\text{dc})}\sigma_Z \\ -H_X - b_{J(\text{dc})}\sigma_Z & i\omega/\gamma - a_{J(\text{dc})}\sigma_Z + \alpha H_Y \end{pmatrix} \begin{pmatrix} \tilde{m}_{2X} \\ \tilde{m}_{2Y} \end{pmatrix} = \begin{pmatrix} -\tilde{a}_{J(\text{ac})}\sigma_X + \tilde{b}_{J(\text{ac})}\sigma_Y \\ -\tilde{a}_{J(\text{ac})}\sigma_Y - \tilde{b}_{J(\text{ac})}\sigma_X \end{pmatrix}, \tag{13}$$

where we neglect higher order terms of α; remember that $a_{J(\text{dc})}$ and $b_{J(\text{dc})}$ are also on the order of α, as mentioned above. The solutions of $\tilde{m}_{2X}$ and $\tilde{m}_{2Y}$ are given by

$$\tilde{m}_{2X} \cong \frac{(i\omega/\gamma)\left(\tilde{a}_{J(\text{ac})}\sigma_X - \tilde{b}_{J(\text{ac})}\sigma_Y\right) - H_Y\left(\tilde{a}_{J(\text{ac})}\sigma_Y + \tilde{b}_{J(\text{ac})}\sigma_X\right)}{\Delta}, \tag{14}$$

$$\tilde{m}_{2Y} \cong \frac{(i\omega/\gamma)\left(\tilde{a}_{J(\text{ac})}\sigma_Y + \tilde{b}_{J(\text{ac})}\sigma_X\right) + H_X\left(\tilde{a}_{J(\text{ac})}\sigma_X - \tilde{b}_{J(\text{ac})}\sigma_Y\right)}{\Delta}, \tag{15}$$

where we introduce

$$\Delta = \left(\frac{\omega}{\gamma}\right)^2 - \left(\frac{\omega_{\text{FMR}}}{\gamma}\right)^2 - i\frac{\omega\delta\omega}{\gamma^2}. \tag{16}$$

The FMR frequency $f_{\text{FMR}} = \omega_{\text{res}}/(2\pi)$ and linewidth $\delta f = \delta\omega/(2\pi)$ are given by

$$f_{\mathrm{FMR}} = \frac{\gamma}{2\pi}\sqrt{\left(H_X + b_{J(\mathrm{dc})}\sigma_Z\right)\left(H_Y + b_{J(\mathrm{dc})}\sigma_Z\right) + \left(a_{J(\mathrm{dc})}\sigma_Z\right)^2} \cong \frac{\gamma}{2\pi}\sqrt{H_X H_Y}$$
$$\cong \frac{\gamma}{2\pi}\sqrt{H_{\mathrm{ext}}(H_{\mathrm{ext}} + 4\pi M)}, \tag{17}$$

$$\delta f = \frac{\gamma}{2\pi}\left[\alpha\left(H_X + H_Y + 2b_{J(\mathrm{dc})}\sigma_Z\right) - 2a_{J(\mathrm{dc})}\sigma_Z\right] \cong \frac{\gamma}{2\pi}\left[\alpha(H_X + H_Y) - 2a_{J(\mathrm{dc})}\sigma_Z\right]$$
$$\cong \frac{2\gamma}{2\pi}\left[\alpha(H_{\mathrm{ext}} + 2\pi M) - a_{J(\mathrm{dc})}\sigma_Z\right]. \tag{18}$$

We remind readers that the spin polarization $\boldsymbol{\sigma}$ points to either x or y direction, whereas the oscillation axis lies in the xy plane, as indicated by supplementary eq. (9), i.e., both θ_σ and θ are $\pi/2$. Then, σ_X defined in the XYZ coordinate becomes zero; see supplementary eq. (11). Therefore, supplementary eq. (14) and (15) are further simplified as

$$\widetilde{m}_{2X} \cong -\frac{(\mathrm{i}\omega/\gamma)\tilde{b}_{J(\mathrm{ac})} + H_Y\tilde{a}_{J(\mathrm{ac})}}{\Delta}\sigma_Y, \tag{19}$$

$$\widetilde{m}_{2Y} \cong \frac{(\mathrm{i}\omega/\gamma)\tilde{a}_{J(\mathrm{ac})} - H_X\tilde{b}_{J(\mathrm{ac})}}{\Delta}\sigma_Y, \tag{20}$$

where $\sigma_Y = -\sin(\varphi - \varphi_\sigma) \cong -\sin(\varphi_H - \varphi_\sigma)$ becomes $|\sigma_Y| = |\sin\varphi_H|$ when $\boldsymbol{\sigma}$ is parallel to the x axis ($\varphi_\sigma = 0, \pi$), whereas $|\sigma_Y| = |\cos\varphi_H|$ when $\boldsymbol{\sigma}$ is parallel to the y axis ($\varphi_\sigma = 2\pi/2, 3\pi/2$).

Section 4.5: FMR spectra formula

Magnetization oscillation can be detected through a direct voltage generated by current-in-plane giant magnetoresistance (CIP-GMR) effect. A model of CIP-GMR is given by

$$R = \frac{R_{\mathrm{P}} + R_{\mathrm{AP}}}{2} - \frac{R_{\mathrm{AP}} - R_{\mathrm{P}}}{2}\mathbf{m}_1 \cdot \mathbf{m}_2, \tag{21}$$

where R_{P} and R_{AP} are resistances when the magnetization alignments are parallel and antiparallel, respectively. We assume that the alternating current is given in the form of $I_{\mathrm{ac}}\sin\omega t$. Then, the direct voltage measured in experiment is given by

$$V = \int_0^T RI_{\mathrm{ac}}\sin\omega t\frac{\mathrm{d}t}{T} = \frac{-\Delta RI_{\mathrm{ac}}}{4}(\mathrm{Re}[\widetilde{m}_{2X}]m_{1X} + \mathrm{Re}[\widetilde{m}_{2Y}]m_{1Y}), \tag{22}$$

where m_{1X} and m_{1Y} are the X and Y components of $\mathbf{m}_1$, whereas $\Delta R = R_{\mathrm{AP}} - R_{\mathrm{P}}$. Since $\mathbf{m}_1$ is parallel to the z axis and $\theta \cong \pi/2$, we notice that $m_{1X} = -\mathrm{sign}(m_{1z})$ and $m_{1Y} = 0$. Substituting supplementary eq. (19) into supplementary eq. (22) therefore, we find that

$$V = \frac{-\Delta R I_{\rm ac}}{4} \mathrm{Re}\left[\frac{(\mathrm{i}\omega/\gamma)\tilde{b}_{J(\mathrm{ac})} + H_Y \tilde{a}_{J(\mathrm{ac})}}{\Delta}\sigma_Y\right] \mathrm{sign}(m_{1z})$$
$$= \frac{-\Delta R I_{\rm ac}}{4} \frac{(f^2 - f_{\rm res}^2)\tilde{\gamma}^2 H_Y \tilde{a}_{J(\mathrm{ac})} - f^2 \delta f \tilde{\gamma} \tilde{b}_{J(\mathrm{ac})}}{(f^2 - f_{\rm res}^2)^2 + (f\delta f)^2} \sigma_Y \mathrm{sgn}(m_{1z}), \qquad (23)$$

where $\tilde{\gamma} = \gamma/(2\pi)$.

Section 4.5: Field representation

Supplementary eq. (23) is represented in terms of the frequency f of the alternating current, whereas the external magnetic field $H_{\rm ext}$ is varied in experiment. Therefore, it is useful to express supplementary eq. (23) in terms of $H_{\rm ext}$. Here, let us study an approximation to transform supplementary eq. (23) into the field representation.

It is useful to note that the experiment is performed near the resonance condition, $f \cong f_{\rm FMR}$. In this case, terms in supplementary eq. (23) can be approximated as

$$\frac{(f^2 - f_{\rm FMR}^2)}{(f^2 - f_{\rm FMR}^2)^2 + (f\delta f)^2} \cong \frac{f - f_{\rm FMR}}{2 f_{\rm res}[(f - f_{\rm FMR})^2 + (\delta f/2)^2]}, \qquad (24)$$

$$\frac{f^2 \delta f}{(f^2 - f_{\rm FMR}^2)^2 + (f\delta f)^2} \cong \frac{\delta f}{4[(f - f_{\rm FMR})^2 + (\delta f/2)^2]}. \qquad (25)$$

Next, let us denote $H_{\rm ext}$ satisfying $f = f_{\rm FMR}$ as $H_{\rm res}$, where f should be regarded as a parameter fixed by experimental circuit, whereas $H_{\rm res}$ is a variable which can be controlled by hand. When $H_{\rm ext}$ is close to $H_{\rm res}$, $f_{\rm FMR}$ can be approximated as

$$f_{\rm FMR}(H_{\rm ext}) \cong f_{\rm FMR}(H_{\rm res}) + \left(\frac{\mathrm{d}f_{\rm FMR}}{\mathrm{d}H_{\rm ext}}\right)_{H_{\rm ext}=H_{\rm res}} (H_{\rm ext} - H_{\rm res}). \qquad (26)$$

Similarly, we define a linewidth W in the field representation through the following relation;

$$\delta f \cong \left(\frac{\mathrm{d}f_{\rm FMR}}{\mathrm{d}H_{\rm ext}}\right)_{H_{\rm ext}=H_{\rm res}} \Delta H. \qquad (27)$$

Then, we notice that supplementary eq. (24) and (25) are further approximated as

$$\frac{(f^2 - f_{\rm FMR}^2)}{(f^2 - f_{\rm FMR}^2)^2 + (f\delta f)^2} \cong \frac{-(H_{\rm ext} - H_{\rm res})}{2 f_{\rm res}\left(\frac{\mathrm{d}f_{\rm FMR}}{\mathrm{d}H_{\rm ext}}\right)_{H_{\rm ext}=H_{\rm res}} [(H_{\rm ext} - H_{\rm res})^2 + (\Delta H/2)^2]}, \qquad (28)$$

$$\frac{f^2 \delta f}{(f^2 - f_{\rm FMR}^2)^2 + (f\delta f)^2} \cong \frac{\Delta H}{4\left(\frac{\mathrm{d}f_{\rm FMR}}{\mathrm{d}H_{\rm ext}}\right)_{H_{\rm ext}=H_{\rm res}} [(H_{\rm ext} - H_{\rm res})^2 + (\Delta H/2)^2]}. \qquad (29)$$

Therefore, the direct voltage measured in experiment is given by

$$V = \frac{-\Delta R I_{\rm ac}}{4\left(\frac{{\rm d}f_{\rm FMR}}{{\rm d}H_{\rm ext}}\right)_{H_{\rm ext}=H_{\rm res}}} \left\{ \frac{(H_{\rm ext} - H_{\rm res})\tilde{\gamma}^2 H_Y \tilde{a}_{J({\rm ac})}}{2f_{\rm res}[(H_{\rm ext} - H_{\rm res})^2 + (\Delta H/2)^2]} - \frac{\Delta H \tilde{\gamma} \tilde{b}_{J({\rm ac})}}{4[(H_{\rm ext} - H_{\rm res})^2 + (\Delta H/2)^2]} \right\} \sigma_Y {\rm sgn}(m_{1z}). \tag{30}$$

Supplementary eq. (30) indicates that the spin-torque FMR spectrum is a linear combination of Lorenzian and anti-Lorenzian functions. In addition to this, because supplementary eq. (30) contains ${\rm sgn}(m_{1z})$, the sign of the V can reverse by reversing the magnetization of CoNi. This is consistent with the spectra shown in Fig.2b. It is worth note that the magnitude of Lorentzian and anti-Lorentzian component changes in the present system. This is because supplementary eq. (30) is proportional to σ_Y consisted of magnetization-independent and magnetization dependent charge-to-spin conversion.

Finally, we derive the resonance linewidth under bias current. Using supplementary eq. (18), (27) and

$$\left(\frac{{\rm d}f_{\rm FMR}}{{\rm d}H_{\rm ext}}\right)_{H_{\rm ext}=H_{\rm res}} \cong \left(\frac{\gamma}{2\pi}\right)^2 \frac{H_{\rm res} + 2\pi M}{f_{\rm res}}, \tag{31}$$

we notice that the linewidth $W = \Delta H/2$ in Eq. (S4-30) becomes

$$W = \frac{2\pi}{\gamma} f_{\rm res} \left(\alpha - \frac{a_{J({\rm dc})}\sigma_Z}{H_{\rm res} + 2\pi M} \right). \tag{32}$$

The strength of the damping-like torque is

$$a_{J({\rm dc})} = \frac{\hbar \tilde{\xi} \eta [I_{\rm dc}/(w d_1)]}{2eMd_2}. \tag{33}$$

where $I_{\rm dc}$ is the total direct current flowing in the x direction, η is the shunting current ratio in the CoNi reference layer, w is the sample width, and d_1 and d_2 are the thicknesses of the CoNi reference layer and Fe-B free layer, respectively. The dimensionless spin-torque efficiency is denoted as $\tilde{\xi}$, which relates to the spin current conductivity ξ defined in the main text via $\xi = \tilde{\xi}\sigma_1$, where σ_1 is the conductivity of CoNi reference layer. In our definition, $\xi_{\rm MD}$ and $\xi_{\rm MI}$ are defined so that the spin torques generated by positive current move the magnetization to ${\rm sign}(m_{1z})x$ and $-y$ directions, respectively. Then, $\sigma_Z = -{\rm sign}(m_{1z})\cos\varphi$ for $\xi_{\rm MD}$ and $\sigma_Z = \sin\varphi$ for $\xi_{\rm MI}$. Thus, supplementary eq. (32) becomes

$$W = \frac{2\pi}{\gamma} f_{\rm res} \left\{ \alpha - \frac{\hbar\eta[\xi_{\rm MI}\sin\varphi - {\rm sign}(m_{1z})\xi_{\rm MD}\cos\varphi] I_{\rm dc}}{2eM\sigma_1 w d_1 d_2 (H_{\rm res} + 2\pi M)} \right\}. \tag{34}$$

We note that the magnetization direction, φ, is parallel to the direction of the external magnetic field. Therefore, the spin-torque conductivities, $\xi_{\rm MD}$ and $\xi_{\rm MI}$, can be evaluated by measuring the spin-torque FMR linewidth under the effect of the external magnetic field pointing in the x and y directions, respectively.

The above formalism is applicable to analyze the spin-torque FMR spectrum in the other geometries by changing the variables such as magnetic field and the spin-polarized directions. For example, a spin-torque FMR spectrum in an in-plane magnetized source layer and a perpendicularly magnetized free layer could be analyzed in a similar way. In such geometry, we can evaluate out-of-plane spin polarized spin current originating from the magnetic-dependent charge-to-spin conversion. We note, however, that the resonance linewidth in perpendicularly magnetized layer is much broader compared to in-plane magnetized system due to its large inhomogeneous broadening [4]. This makes the quantitative evaluation of the damping modulation difficult in this geometry. In addition, it is technically difficult to change the magnetization direction of the in-plane magnetized source layer, which makes it difficult to study the dependence of the spin polarization on the magnetization direction from a single device. Regarding these facts, we performed spin-torque FMR measurement in a perpendicularly magnetized source layer and an in-plane magnetized free layer to identify the existence and symmetry of the magnetic-dependent charge-to-spin conversion induced spin current.

Supplementary Note 5:
Theoretical formula of spin current conductivity

In this note, we provide the physical picture of the spin Hall precession effect we propose in this work and show the derivation of the theoretical formula of spin current conductivity via the mechanism. The formulation is based on our previous work [5,6], which combines the diffusive spin transport theory in bulk [7] and the spin-dependent Landauer theory of interface transport [8]. In addition, we use the source terms of spin current generated in ferromagnet proposed in Supplementary ref. 9. We emphasize that multiple mechanisms can generate bulk spin current, as written in the main text. The spin Hall precession effect discussed below relates to the extrinsic mechanism of the spin Hall effect, such as skew scattering, where the spin transport could be diffusive due to the scattering and the spin polarization perpendicular to the local magnetization experiences dephasing [7]. On the other hand, the spin current generated by the intrinsic mechanism is carried by electrons in perturbated eigenstates with same wave vector and does not experience the dephasing [10]. The first principles calculations will be necessary to evaluate bulk spin current [10,11].

The system consists of two ferromagnets, F_1 and F_2, and nonmagnetic spacer, N. The F_1, F_2, and N layers correspond to CoNi, Fe-B, and Cu in the main text, respectively (Supplementary figure 6). Throughout this section, when it is necessary, we add the suffixes, F_1, F_2, and N, to quantities related to these layers, whereas the suffixes (or superscripts) such as F_1/N are used to distinguish quantities related to the ferromagnetic/nonmagnetic interface. The suffixes 1 and 2 are also used to quantities related to F_k (k=1,2) layers and/or F_k/N interface. For example, the unit vector pointing in the magnetization direction of F_k layer is denoted as $\mathbf{m}_k$. The z axis is normal to the film plane, whereas the x axis is parallel to the in-plane current direction. The words “longitudinal” and “transverse” are used to distinguish the directions parallel and perpendicular to the local magnetization, respectively.

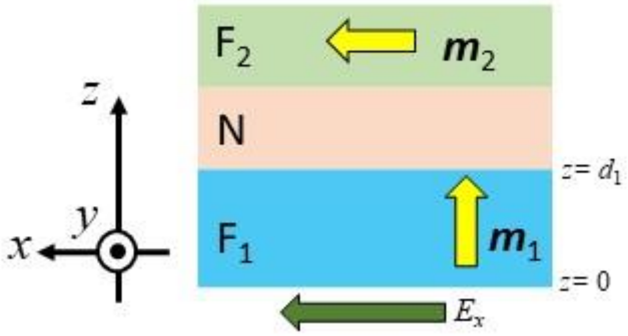

Supplementary Figure 6: Schematic for the theoretical model.

Section 5.1: Spin transport theory in F_1 layer

Let us first consider the spin diffusion in F_1 layer. The spin accumulation $\boldsymbol{\mu}_{F_1}$ can be decomposed into the longitudinal and transverse components with respect to $\mathbf{m}_1$ as $\boldsymbol{\mu}_{F_1}^{L} = (\mathbf{m}_1 \cdot \boldsymbol{\mu}_{F_1})\mathbf{m}_1$ and $\boldsymbol{\mu}_{F_1}^{T} = \mathbf{m}_1 \times (\boldsymbol{\mu}_{F_1} \times \mathbf{m}_1)$. The longitudinal spin accumulation relates to the electrochemical potential $\bar{\mu}_s$ of spin-s electrons ($s = \uparrow, \downarrow$) via $\boldsymbol{\mu}_{F_1}^{L} = (\bar{\mu}_\uparrow - \bar{\mu}_\downarrow)\mathbf{m}_1$, and obeys the diffusion equation [12],

$$\frac{\mathrm{d}^2 \boldsymbol{\mu}_{F_1}^{L}}{\mathrm{d}z^2} = \frac{\boldsymbol{\mu}_{F_1}^{L}}{\lambda_{\mathrm{sd1}}^2}, \tag{35}$$

where λ_{sd1} is the spin diffusion length of the F_1 layer. The electrochemical potential relates to the longitudinal spin current via

$$\mathbf{m}_1 \cdot \mathbf{I}_{\mathrm{s}} = -\frac{\mathrm{d}}{\mathrm{d}z}\frac{\hbar S}{2e^2}(\sigma_\uparrow \bar{\mu}_\uparrow - \sigma_\downarrow \bar{\mu}_\downarrow), \tag{36}$$

where S is the cross-section area of F_1/N interface, whereas σ_s is the conductivity of spin-s electrons. The elementary charge is defined as $e > 0$.We assume that the F_1 layer locates in the region of $0 \le z \le d_1$, where d_1 is the thickness of the F_1 layer. The open boundary condition is applied to $z = 0$, whereas the total spin current at the F_1/N interface at $z = d_1$ is denoted as $\mathbf{I}_{\mathrm{s}}^{F_1/N}$. We note that $\mathbf{I}_{\mathrm{s}}^{F_1/N}$ will be given by the Landauer formula, as discussed below. For a while, let us regard $\mathbf{I}_{\mathrm{s}}^{F_1/N}$ as an integral constant of supplementary eq. (35). The solution of supplementary eq. (35) with supplementary eq. (36) is then given by

$$\mathbf{m}_1 \cdot \boldsymbol{\mu}_{F_1}^{L} = -\frac{4\pi}{g_{\mathrm{sd1}} \sinh(d_1/\lambda_{\mathrm{sd1}})} \mathbf{m}_1 \cdot \mathbf{I}_{\mathrm{s}}^{F_1/N} \cosh\left(\frac{z}{\lambda_{\mathrm{sd1}}}\right), \tag{37}$$

where we introduce

$$g_{\mathrm{sd1}} = \frac{h(1-\beta_1^2)S}{2e^2 \rho_1 \lambda_{\mathrm{sd1}}}, \tag{38}$$

where $\rho_1 = 1/\sigma_1$ is the resistivity of the F_1 layer, whereas $\beta = (\sigma_\uparrow - \sigma_{\uparrow\downarrow})/(\sigma_\uparrow + \sigma_{\uparrow\downarrow})$ is the spin polarization of the bulk conductivity.

On the other hand, the transverse spin accumulation obeys [6]

$$\frac{\mathrm{d}^2 \boldsymbol{\mu}_{F_1}^{T}}{\mathrm{d}z^2} = \frac{\boldsymbol{\mu}_{F_1}^{T}}{\lambda_{\mathrm{t1}}^2} + \frac{\boldsymbol{\mu}_{F_1}^{T} \times \mathbf{m}_1}{\lambda_{J1}^2}, \tag{39}$$

where λ_{t1} is the spin diffusion length of the transverse spin accumulation. For simplicity, we assume that the value of the spin polarization of the diffusion constant β' [7] is close to that of the conductivity, β. Then, we find that $\lambda_{\mathrm{t1}} = \lambda_{\mathrm{sd1}}/\sqrt{1-\beta_1^2}$. On the other hand, λ_{J1} is a length scale characterizing the precession of the transverse spin around the magnetization $\mathbf{m}_1$ via exchange interaction [7]. We note that the transverse spin current in Supplementary refs. 5-7 relates to the transverse spin accumulation as

$$\mathbf{m}_1 \times (\mathbf{I}_\mathrm{s} \times \mathbf{m}_1) = -\frac{\mathrm{d}}{\mathrm{d}z}\frac{\hbar S \sigma_{\uparrow\downarrow}}{2e^2}\boldsymbol{\mu}_{\mathrm{F}_1}^{\mathrm{T}}, \quad (40)$$

where $\sigma_{\uparrow\downarrow}$ is the conductivity of the transverse spin current, which becomes $\sigma_1/2$ under the assumption of $\beta' \approx \beta$ [3]. Since we are interested in the spin current generation in bulk ferromagnet, however, we add the source terms proposed in Supplementary ref. 7. Therefore, supplementary eq. (40) is replaced by

$$\mathbf{m}_1 \times (\mathbf{I}_\mathrm{s} \times \mathbf{m}_1) = -(1 - \theta_\mathrm{p}\mathbf{m}_1 \times)\frac{\mathrm{d}}{\mathrm{d}z}\frac{\hbar S \sigma_1}{4e^2}\boldsymbol{\mu}_{\mathrm{F}_1}^{\mathrm{T}} + \frac{\hbar S \sigma_1}{2e}(\vartheta_y \mathbf{t}_2 - \vartheta_x \mathbf{m}_1 \times \mathbf{t}_2)E_x, \quad (41)$$

where $\mathbf{t}_2$ is the unit vector in spin space orthogonal to $\mathbf{m}_1$. We also introduce a unit vector $\mathbf{t}_1$, which satisfies $\mathbf{t}_1 \times \mathbf{t}_2 = \mathbf{m}_1$. The electric field applied in the x direction is denoted as E_x. The parameter θ_p is introduced in Supplementary ref. 9. The source terms are characterized by two spin Hall angles, ϑ_x and ϑ_y. We emphasize that the vectors $\mathbf{t}_1$, $\mathbf{t}_2$, and $\mathbf{m}_1$ are defined in spin space. In the present study, however, since $\mathbf{m}_1$ in CoNi is perpendicularly magnetized and therefore points to the z direction, we can regard $\mathbf{t}_1$ and $\mathbf{t}_2$ as the unit vectors pointing in the x and y directions in spatial space, and the origins of the source terms proportional to ϑ_x and ϑ_y are regarded as the spin swapping and spin Hall effects, respectively [8]. In further detail, $\mathbf{m}_1$ in our experiment points to either positive or negative z direction. Accordingly, $\mathbf{t}_1 = -\mathbf{m}_1 \times \mathbf{t}_2$ points to either positive or negative x direction, depending on the sign of m_{1z}, where $\mathbf{t}_2 = \mathbf{e}_y$ for the spin Hall effect. Note that, if the magnetization has a projection to the y direction, the source term of the spin Hall effect $\vartheta_y \mathbf{t}_2$ has a projection to the magnetization direction. Such a term should be included the definition of the longitudinal spin current given by supplementary eq. (36). In addition, if we consider another source term such as anomalous Hall effect and/or anisotropic magnetoresistance [13], it should be included in supplementary eq. (36). In the following, however, we focus on the experimental situation in the main text, where the magnetization points to the z direction, as mentioned above. In this case, supplementary eq. (41) is enough to describe the spin current generation in bulk ferromagnet. The solution of supplementary eq. (39) with supplementary eq. (41) are given by [6]

$$\mu_+ = \frac{-4\pi}{g_{\mathrm{t}1}\sinh(d_1/\ell_1)}\left[I_{\mathrm{T}+}\cosh\left(\frac{z-d_1}{\ell_1}\right) + \left(I_{\mathrm{T}+}^{\mathrm{F}_1/\mathrm{N}} - I_{\mathrm{T}+}\right)\cosh\left(\frac{z}{\ell_1}\right)\right] \quad (42)$$

where $\mu_+ = \mathbf{t}_1 \cdot \boldsymbol{\mu}_{\mathrm{F}_1}^{\mathrm{T}} + \mathrm{i}\mathbf{t}_2 \cdot \boldsymbol{\mu}_{\mathrm{F}_1}^{\mathrm{T}}$ and $I_{\mathrm{T}+}^{\mathrm{F}_1/\mathrm{N}} = \mathbf{t}_1 \cdot \mathbf{I}_\mathrm{s}^{\mathrm{F}_1/\mathrm{N}} + \mathrm{i}\mathbf{t}_2 \cdot \mathbf{I}_\mathrm{s}^{\mathrm{F}_1/\mathrm{N}}$ are introduced, for simplicity. We also define [6,7,9]

$$g_{\mathrm{sd}1} = \frac{(1-\mathrm{i}\theta_\mathrm{p})hS}{2e^2\rho_1\ell_1}, \quad (43)$$

$$\frac{1}{\ell_1} = \sqrt{\frac{1}{1-\mathrm{i}\theta_\mathrm{p}}\left(\frac{1}{\lambda_{\mathrm{t}1}^2} - \mathrm{i}\frac{1}{\lambda_{J1}^2}\right)}, \quad (44)$$

and $I_{\mathrm{T}+} = \hbar(\vartheta_x + \mathrm{i}\vartheta_y)\sigma_1 E_x S/(2e)$.

Section 5.2: Spin transport theory across F_1/N interface

In this section, we apply spin-dependent Landauer formula to the boundary conditions of supplementary eq. (35) and (40). The spin-dependent Landauer formula gives the relation between the ferromagnetic/nonmagnetic interface and spin accumulations as [8]

$$\mathbf{I}_{\mathrm{s}}^{\mathrm{F_1/N}} = \frac{-1}{4\pi}\left[\frac{(1-\gamma_1^2)g_1}{2}\mathbf{m}_1\cdot(\boldsymbol{\mu}_{\mathrm{N}}-\boldsymbol{\mu}_{\mathrm{F_1}})\mathbf{m}_1 + g_{\mathrm{r1}}\mathbf{m}_1\times(\boldsymbol{\mu}_{\mathrm{N}}\times\mathbf{m}_1) + g_{\mathrm{i1}}\boldsymbol{\mu}_{\mathrm{N}}\times\mathbf{m}_1 \right.$$
$$\left. - t_{\mathrm{r1}}\mathbf{m}_1\times(\boldsymbol{\mu}_{\mathrm{F_1}}\times\mathbf{m}_1) - t_{\mathrm{i1}}\boldsymbol{\mu}_{\mathrm{F_1}}\times\mathbf{m}_1\right], \tag{45}$$

where $g_1 = (g_1^{\uparrow}+g_1^{\downarrow})$ is the dimensionless interface conductivity, whereas $\gamma = (g_1^{\uparrow}-g_1^{\downarrow})/(g_1^{\uparrow}+g_1^{\downarrow})$ is its spin polarization. The interface resistance at the F_1/N interface is $r_1 = (h/e^2)S/g_1$. The real and imaginary parts of the dimensionless mixing conductance [7] are denoted as g_{r1} and g_{i1}, respectively, whereas the real and imaginary parts of the dimensionless transmission mixing conductance, introduced in Supplementary refs. [5,6], are t_{r1} and t_{i1}, respectively. The spin accumulation in N layer is $\boldsymbol{\mu}_{\mathrm{N}}$. Substituting supplementary eq. (37) and (42) into supplementary eq. (45), we find that supplementary eq. (45) can be rewritten as

$$\mathbf{I}_{\mathrm{s}}^{\mathrm{F_1/N}} = \mathbf{I}_{s\mathrm{T}} - \frac{1}{4\pi}\left[g_1^{*}(\mathbf{m}_1\cdot\boldsymbol{\mu}_{\mathrm{N}})\mathbf{m}_1 + \tilde{g}_{\mathrm{r1}}\mathbf{m}_1\times(\boldsymbol{\mu}_{\mathrm{N}}\times\mathbf{m}_1) + \tilde{g}_{\mathrm{i1}}\boldsymbol{\mu}_{\mathrm{N}}\times\mathbf{m}_1\right], \tag{46}$$

where we introduce [S6]

$$\frac{1}{g_1^{*}} = \frac{2}{(1-\gamma_1^2)g_1} + \frac{1}{g_{\mathrm{sd1}}\tanh(d_1/\lambda_{\mathrm{sd1}})}, \tag{47}$$

$$\tilde{g}_{\mathrm{r1}} = \frac{A_1 g_{\mathrm{r1}} + B_1 g_{\mathrm{i1}}}{A_1^2+B_1^2},\quad \tilde{g}_{\mathrm{i1}} = \frac{A_1 g_{\mathrm{i1}} - B_1 g_{\mathrm{r1}}}{A_1^2+B_1^2}, \tag{48}$$

$$A_1 = 1 + \mathrm{Re}\left[\frac{t_{\mathrm{r1}} - \mathrm{i}t_{\mathrm{i1}}}{g_{\mathrm{t1}}\tanh(d_1/\ell_1)}\right],\quad B_1 = -\mathrm{Im}\left[\frac{t_{\mathrm{r1}} - \mathrm{i}t_{\mathrm{i1}}}{g_{\mathrm{t1}}\tanh(d_1/\ell_1)}\right]. \tag{49}$$

The source term $\mathbf{I}_{s\mathrm{T}}$ is given by

$$\mathbf{I}_{s\mathrm{T}} = \mathrm{Re}\left[\frac{\tilde{t}_{\mathrm{r1}} - \mathrm{i}\tilde{t}_{\mathrm{i1}}}{g_{\mathrm{t1}}} I_{\mathrm{T+}}\tanh\left(\frac{d_1}{2\ell_1}\right)\right]\mathbf{t}_1 + \mathrm{Im}\left[\frac{\tilde{t}_{\mathrm{r1}} - \mathrm{i}\tilde{t}_{\mathrm{i1}}}{g_{\mathrm{t1}}} I_{\mathrm{T+}}\tanh\left(\frac{d_1}{2\ell_1}\right)\right]\mathbf{t}_2, \tag{50}$$

where the normalized transmission mixing conductance, $\tilde{t}_{\mathrm{r1}}$ and $\tilde{t}_{\mathrm{i1}}$ are defined in a similar manner of the normalized mixing conductance defined in supplementary eq. (48).

Section 5.3: Spin current generated at F/N interface

Before proceeding further calculation of spin current conductivity, let us discuss spin-current generation at ferromagnetic/nonmagnetic interface. Recently, it was proposed that the interface spin-orbit interaction generate spin current flowing transverse to the direction of the external electric field [14,15]. Typically, spin current generated at ferromagnetic/nonmagnetic interface has two

polarizations in the directions of $\boldsymbol{j}_{\mathrm{e}} \times \mathbf{e}_z$ and $\mathbf{m} \times (\boldsymbol{j}_{\mathrm{e}} \times \mathbf{e}_z)$, where $\boldsymbol{j}_{\mathrm{e}}$ is the electric current density. The interface spin-orbit interaction causes spin-flip scattering, which is not included in the spin-dependent Landauer formula [8]. An extension to the Landauer formula was then proposed in, for example. Supplementary ref. 16. In a simplified case, the nonconservative spin transport might be described by introducing renormalized interface conductance [14]. In addition, it should be remembered that spin-current generation at the interface, such as spin pumping [17], has been investigated even only the spin-conservative transport is taken into account. Let us denote spin current generated at ferromagnetic/nonmagnetic interface as

$$\mathbf{I}_{\mathrm{s}}^{\mathrm{I}} = \frac{1}{4\pi}\left(\Gamma_x \mathbf{t}_1 + \Gamma_y \mathbf{t}_2\right), \tag{51}$$

where Γ_x and Γ_y characterize the amount of the spin current generated at the interface. For example, in the case of spin pumping [S17], $\Gamma_x = \hbar g_{\mathrm{r}1}|\mathbf{m}_1 \times \dot{\mathbf{m}}_1|$ and $\Gamma_y = -\hbar g_{\mathrm{i}1}|\dot{\mathbf{m}}_1|$ when we choose the coordinate as $\mathbf{t}_1 = \mathbf{m}_1 \times \dot{\mathbf{m}}_1/|\mathbf{m}_1 \times \dot{\mathbf{m}}_1|$ and $\mathbf{t}_2 = -\dot{\mathbf{m}}_1/|\dot{\mathbf{m}}_1|$. In fact, assuming that the source term is spin pumping, the following formulation reproduces the results developed in Supplementary refs. 5 and 6. We note that the spin current given by supplementary eq. (51) should be added to the boundary conditions of the diffusion equations, supplementary eq. (35) and (40). Therefore, the spin current $\mathbf{I}_{\mathrm{s}}^{\mathrm{F}_1/\mathrm{N}}$ in supplementary eq. (37) and (42) should be replaced by $\mathbf{I}_{\mathrm{s}}^{\mathrm{I}} + \mathbf{I}_{\mathrm{s}}^{\mathrm{F}_1/\mathrm{N}}$. Then, we notice that the interface-driven spin current, supplementary eq. (51), give an additional source term

$$\mathbf{I}_{\mathrm{s}}^{\mathrm{Is}} = \frac{1}{4\pi}\left(\tilde{\Gamma}_x \mathbf{t}_1 + \tilde{\Gamma}_y \mathbf{t}_2\right), \tag{52}$$

where

$$\tilde{\Gamma}_x = \frac{A_1\Gamma_x - B_1\Gamma_y}{A_1^2 + B_1^2}, \quad \tilde{\Gamma}_y = \frac{A_1\Gamma_y + B_1\Gamma_x}{A_1^2 + B_1^2}. \tag{53}$$

The total source term become $\mathbf{I}_{\mathrm{s}}^{\mathrm{source}} = \mathbf{I}_{\mathrm{sT}} + \mathbf{I}_{\mathrm{s}}^{\mathrm{Is}}$.

Section 5.4: Spin current conductivity

We note that the spin diffusion equation in bulk and interface transport studied in Secs. 5.2 and 5.3 are also applied to F_2 layer and F_2/N interface. We assume that the bulk and interface source terms exist F_1 layer and F_1/N interface only, for simplicity. Note that the nonmagnetic spacer used in our experiment, Cu, has long spin diffusion length compared to its thickness. Therefore, we assume that the total spin current is conserved in N layer. The condition can be expressed as

$$\begin{aligned} &\mathbf{I}_{\mathrm{s}}^{\mathrm{source}} - \frac{1}{4\pi}\left[g_1^*(\mathbf{m}_1 \cdot \boldsymbol{\mu}_{\mathrm{N}})\mathbf{m}_1 + \tilde{g}_{\mathrm{r}1}\mathbf{m}_1 \times (\boldsymbol{\mu}_{\mathrm{N}} \times \mathbf{m}_1)\right] \\ &\qquad - \frac{1}{4\pi}\left[g_2^*(\mathbf{m}_2 \cdot \boldsymbol{\mu}_{\mathrm{N}})\mathbf{m}_2 + \tilde{g}_{\mathrm{r}2}\mathbf{m}_2 \times (\boldsymbol{\mu}_{\mathrm{N}} \times \mathbf{m}_2)\right] = \mathbf{0}, \end{aligned} \tag{54}$$

where we neglect $\tilde{g}_{\mathrm{i}1}$ and $\tilde{g}_{\mathrm{i}2}$ because they are small compared to $\tilde{g}_{\mathrm{r}1}$ and $\tilde{g}_{\mathrm{r}2}$, except very thin

limit of the ferromagnetic thickness, due to the relation $g_{\mathrm{r1}} \gg |g_{\mathrm{i1}}|$. Solving supplementary eq. (54) with respect to $\boldsymbol{\mu}_{\mathrm{N}}$, the spin-transfer torque acting on the magnetization in F_2 layer is obtained as

$$\frac{\mathrm{d}\mathbf{m}_2}{\mathrm{d}t} = \frac{\gamma_0}{MV}\mathbf{m}_2 \times \left(\mathbf{I}_{\mathrm{s}}^{\mathrm{F}_2/\mathrm{N}} \times \mathbf{m}_2\right) = -\frac{\gamma_0 \tilde{g}_{\mathrm{r2}}}{4\pi MV}\mathbf{m}_2 \times (\boldsymbol{\mu}_{\mathrm{N}} \times \mathbf{m}_2), \tag{55}$$

where γ_0, M, and V are the gyromagnetic ratio, saturation magnetization, and volume of the F_2 layer, respectively. We note that supplementary eq. (55) can be expressed in a form of

$$\frac{\mathrm{d}\mathbf{m}_2}{\mathrm{d}t} = -\frac{\gamma_0 \hbar j}{2eMd_2}\mathbf{m}_2 \times \left[\left(\tilde{\xi}_z \mathbf{m}_1 + \tilde{\xi}_x \mathbf{t}_1 + \tilde{\xi}_y \mathbf{t}_2\right) \times \mathbf{m}_2\right], \tag{56}$$

where j is the electric current density flowing in the F_1 layer in the x direction, whereas d_2 is the thickness of F_2 layer. The dimensionless effective spin Hall angles, $\tilde{\xi}_z$, $\tilde{\xi}_x$, and $\tilde{\xi}_y$, depend on the source terms, ϑ_x, ϑ_y, Γ_x, and Γ_y, as well as the magnetization directions. Their general expressions can be obtained from supplementary eq. (54) directly. The explicit expressions are, however, tedious. Since we are interested in spin-torque FMR measurement, where points $\mathbf{m}_2$ to the in-plane direction, let us focus on such a situation only. In addition, we assume that $\vartheta_x = 0$ and $\vartheta_y \neq 0$ because we are interested in the spin Hall effect in the F_1 layer. In spin-torque FMR measurement, $\tilde{\xi}_{x(y)}$ is estimated from the modulation of the spectrum linewidth when the external magnetic field points to the $x(y)$ direction. In this case, $\tilde{\xi}_{x(y)}$ are given by

$$\tilde{\xi}_x = \frac{A_1\Theta_x - B_1\Theta_y}{A_1^2 + B_1^2}\frac{\tilde{g}_{\mathrm{r2}}}{\tilde{g}_{\mathrm{r1}} + g_2^*} - \mathrm{Im}\left[\frac{\tilde{t}_{\mathrm{r1}} - \mathrm{i}\tilde{t}_{\mathrm{i1}}}{g_{\mathrm{t1}}}\tanh\left(\frac{d_1}{2\ell_1}\right)\right]\frac{\tilde{g}_{\mathrm{r2}}}{\tilde{g}_{\mathrm{r1}} + g_2^*}\vartheta_y, \tag{57}$$

$$\tilde{\xi}_y = \frac{A_1\Theta_y + B_1\Theta_x}{A_1^2 + B_1^2}\frac{\tilde{g}_{\mathrm{r2}}}{\tilde{g}_{\mathrm{r1}} + g_2^*} + \mathrm{Re}\left[\frac{\tilde{t}_{\mathrm{r1}} - \mathrm{i}\tilde{t}_{\mathrm{i1}}}{g_{\mathrm{t1}}}\tanh\left(\frac{d_1}{2\ell_1}\right)\right]\frac{\tilde{g}_{\mathrm{r2}}}{\tilde{g}_{\mathrm{r1}} + g_2^*}\vartheta_y, \tag{58}$$

where we introduce $\Theta_{x(y)}$ as $\Gamma_{x(y)} = 4\pi S\Theta_{x(y)}[\hbar j/(2e)]$. We note that the spin conductivities, ξ_{MD} and ξ_{MI} in the main text, are related to $\tilde{\xi}_x$ and $\tilde{\xi}_y$ defined by torque formula, supplementary eq. (56), via

$$\xi_{\mathrm{MD}} = -\tilde{\xi}_x \sigma_1, \tag{59}$$

$$\xi_{\mathrm{MI}} = \tilde{\xi}_y \sigma_1, \tag{60}$$

whereas $\mathbf{t}_1$ and $\mathbf{t}_2$ in supplementary eq. (56) are replaced by $\mathrm{sign}(m_{1z})\mathbf{e}_x$ and $\mathbf{e}_y$, respectively. Note that the bulk contribution to ξ_{MD} is finite even in the case of $\vartheta_x = 0$ because the spin current generated by the spin Hall effect, with the spin Hall angle ϑ_y, changes its direction due to the precession around the magnetization. This is the spin Hall precession effect we propose to generate magnetic-dependent spin current in bulk.

Section 5.5: Dependence of spin current conductivity on thickness of source ferromagnet

Supplementary figures 7(a) and 7(b) show examples of spin conductivities, ξ_{MD} and ξ_{MI}, as a function of the thickness of F_1 layer. For simplicity, we assume that the values of the parameters, except resistivity, between F_1 and F_2 are identical; see supplementary table 1 below, where typical

values are derived from experiments and theories [5-8,17], except λ_J, ϑ_y, Θ_x and Θ_y, which are determined to minimize error between experiment and theory. The solid lines in Supplementary fig. 7 show the total spin conductivities given by supplementary eq. (59) and (60). In addition, we divide them into bulk and interface contributions, proportional to $\Theta_{x,y}$ and ϑ_y, respectively [see also supplementary eq. (57) and (58)] and show these contributions by using dashed and dotted lines. We note that both the interface and bulk contributions show nonmonotonic dependences on d_1. A similar behavior was observed in Supplementary ref. 9. This is due to the precession of the spin polarization around the magnetization, which is described by the last term in supplementary eq. (39). Accordingly, the spin conductivities show weak oscillation before saturation.

We also note that the interface contributions immediately saturate with increasing the thickness, whereas the bulk contributions gradually saturate. The difference comes from the dependences of the amounts of the spin current generated at interface and inside bulk. The source term of the interface-driven spin current is independent of the thickness of F_1 layer. Therefore, the dependence of the interface contribution on d_1 solely comes from the spin diffusion after the source spin current is generated. On the other hand, the amount of the spin current generated inside the bulk depends on the ferromagnetic thickness. Thus, not only the spin diffusion but also the generation of the spin current depend on d_1 in the bulk contribution. These differences give the different thickness dependences of the interface and bulk contributions. Mathematically, the difference is given by the factor $\tanh[d_1/(2\ell_1)]$. Due to its fast saturation, the interface contribution is approximately independent of the ferromagnetic thickness. Therefore, the gradual change of the spin current conductivity can be regarded as an evidence of the existence of spin-current generation inside bulk.

Supplementary table 1. The values of fixed parameters and fitting parameters

Resistivity of CoNi (Ωnm)	200
Resistivity of Fe-B (Ωnm)	1100
Spin polarization of conductivity, β	0.50
Longitudinal conductance, $(1-\gamma^2)g/(2S)$ (nm^{-2})	20
Spin precession angle, θ_p	0
Spin diffusion length, λ_sd (nm)	2.00
Spin coherence length, λ_J (nm) [fitting parameter]	1.75
Real and imaginary parts of mixing conductance, $g_{\mathrm{r1(i1)}}/S$ (nm^{-2})	16 (0)
Real and imaginary parts of transmission mixing conductance, $t_{\mathrm{r1(i1)}}/S$ (nm^{-2})	4 (0)
Thickness of Fe-B, d_2 (nm)	1.3
Bulk spin Hall angle, ϑ_y [fitting parameter]	0.158
Interface spin Hall angles, $\Theta_{x(y)}$ [fitting parameter]	0.005 (0.022)

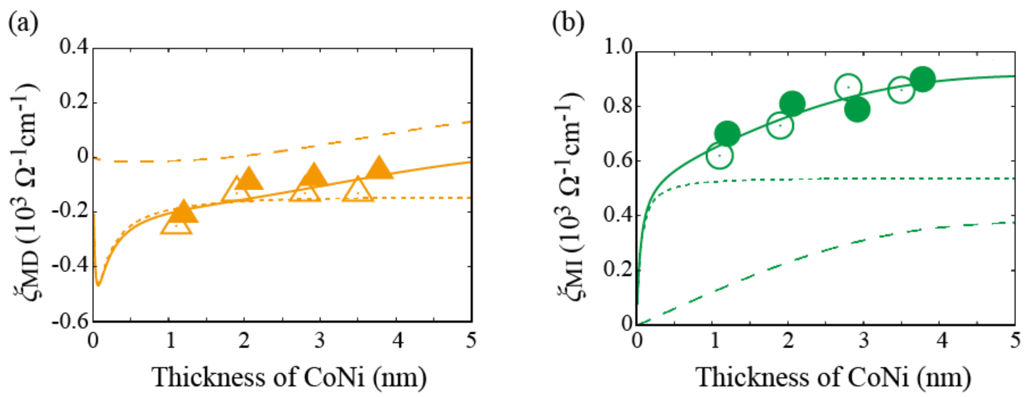

Supplementary figure 7: Detail fitting result of thickness dependence of ξ_{MD} and ξ_{MI}. Dependences of spin conductivities, **a** ξ_{MD} and **b** ξ_{MI}, on the thickness of F_1 layer, d_1, are shown by solid lines. The interface contributions, proportional to $\Theta_{x,y}$ and bulk contributions, proportional to ϑ_y, are also shown by dotted and dashed line, respectively. The experimental results are added as guide for eyes.

Supplementary Note 6:
Magnetic-dependent Charge-to-spin conversion under $+M_{PML}$ and $-M_{PML}$ state

Supplementary figure 8 shows the PML thickness dependence of ξ_{MD} and ξ_{MI} under $+M_{PML}$ and $-M_{PML}$ state using series A samples. As mentioned in the main text, the sign of ξ_{MD} is reversed as the magnetization of PML is switched from $+M_{PML}$ to $-M_{PML}$ state. Meanwhile, there is no significant difference in magnitude of ξ_{MD} between two states.

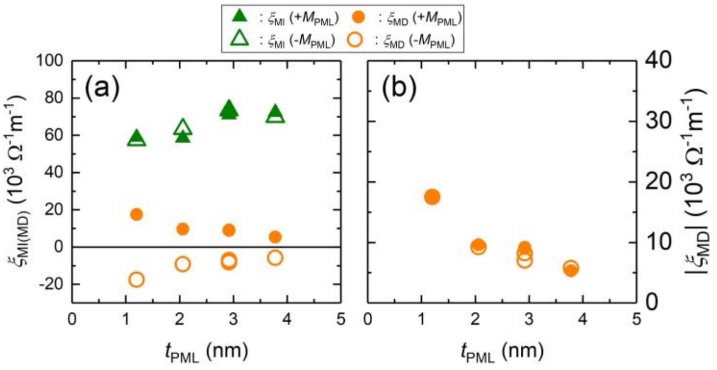

Supplementary figure 8: Charge-to-spin conversion under two magnetic states. a Thickness dependence of spin current conductivity ξ_{MI} (green) and ξ_{MD} (orange) under $+M_{PML}$ and $-M_{PML}$ state (solid and open symbol respectively). **b** thickness dependence of absolute value of ξ_{MD} under $+M_{PML}$ and $-M_{PML}$ state. The data is obtained from series A samples.

Supplementary Note 7:
Charge-to-spin conversion in other PML materials

Thickness dependence of ξ_{MI} and ξ_{MD} was investigated using perpendicularly magnetized ferromagnetic materials different from Co/Ni multilayers used in main text. Here, we used Co/Pt multilayer system which is also known to exhibit strong perpendicular magnetic anisotropy [18]. The detailed stack structure is the following; Ta-B (3.0) / Ru (2.0) / Ir (1.5) / [Co (0.4) / Pt (0.4)]$_n$ / Co (0.4) / Cu (3.0) / $Fe_{75}B_{25}$ (1.3) / MgO / TaO_y (thickness in nm). Here n represents the repetition number (n = 1 ~ 7). The PML thickness dependence of ξ_{MI} and ξ_{MD} are shown in supplementary figure 9(b). As in the case of Co/Ni multilayer, ξ_{MI} shows monotonic increase with t_{PML}, while the ξ_{MD} rapidly decreases with t_{PML}. These tendencies are similar to those observed in Co/Ni multilayers case in the main text, indicating that both interfacial and bulk contributions of charge-to-spin conversion exist in Co/Pt multilayer too. The values of ξ_{MI} and ξ_{MD} in Co/Pt multilayer are, however, smaller than those in Co/Ni multilayer case. In addition, rapid saturations of the spin conductivities imply that the spin coherence length λ_J of Co/Pt multilayer is shorter than that of Co/Ni multilayer.

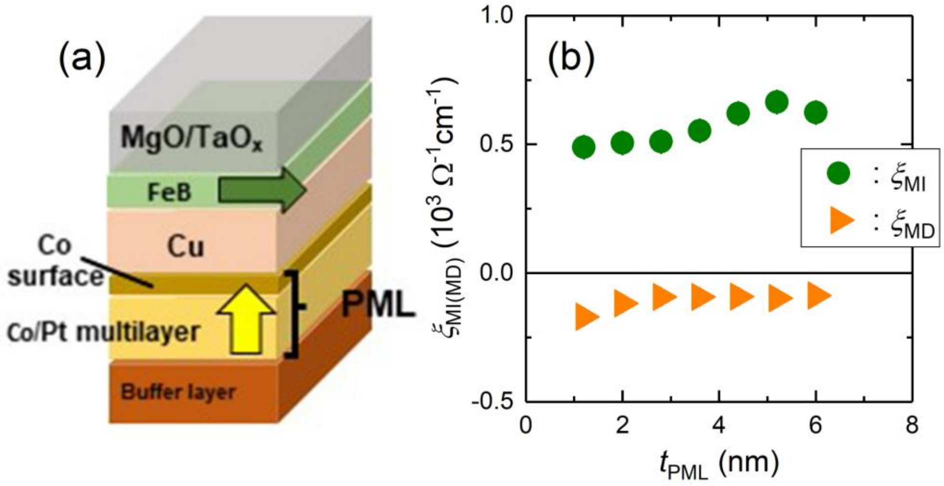

Supplementary figure 9: Charge-to-spin conversion in Co/Pt multilayer spin source. a Schematic view of the tri-layer structure **b** PML thickness of ξ_{MI} (green) and ξ_{MD} (orange) where

Supplementary Not8:
Spin-to-Charge conversion in Co/Ni multilayer

In this section, we demonstrate the inverse process of charge-to-spin conversion (i.e., spin-to-charge conversion), which arise from the Onsager's reciprocity to check the robusticity of our observed results. We used spin pumping method [19-21] to inject pure spin current to Co/Ni multilayer we are focusing on.

Supplementary figure 10a shows the schematic of the device used in the spin pumping experiments. We used Series C samples which were patterned into strips with 200 μm length and 5 μm width dimensions. The coplanar waveguide consisting of Cr(5nm)/Au(200nm) is integrated in the sample where the microstrip is placed in the gap between the signal and ground line of the waveguide. 120 nm SiO_2 insulating layer is deposited between the micro-stripes and the wave guide for separation. The Fe-B layer in the tri-layer structure acts as a spin pumping source, in which ferromagnetic resonance is excited by the rf out-of-plane Oersted field from the waveguide. This spin-pumping induced pure spin current flows into the Co/Ni multilayer and transverse charge current is generated via spin-to-charge conversion. The converted charge current can be detected as an electromotive force V_{elec} of the microstrips. Supplementary figure 10b and 10c shows the measured V_{elec} under external in-plane field applied along φ=90 degree and φ= 0 degree respectively. The figure shows measurement of Series C sample with Ni concentration of 69% and the Co/Ni multilayer is magnetized along +z direction (+M_{PML} state). In both spectra, V_{elec} shows Lorentzian-like peaks with opposite polarity near positive and negative resonance field of Fe-B layer (near ±65 mT) indicating an electromotive force derived from spin-to-charge conversion in Co/Ni multilayer. The difference in the magnitude of V_{elec} indicates a presence of symmetric thermo-electric background [20,21]. Therefore, the electromotive force generated from spin-to-charge conversion (V_{SC}) can be obtained by $V_{\mathrm{SC}} = [V_{\mathrm{elec}}(-H_{\mathrm{res}}) - V_{\mathrm{elec}}(+H_{\mathrm{res}})]/2$. In-plane angular dependence of V_{SC} under +M_{PML} (solid red circles) and -M_{PML} state (open blue circles) is shown in supplementary figure 10c. Considering a magnetic-independent and magnetic-dependent spin-to-charge conversion under spin current flowing out-of-plane direction, V_{SC} can be expressed as follow;

$$V_{\mathrm{SC}} \propto -c[\xi_{\mathrm{MI}} \sin\varphi + \mathrm{sgn}(M_z^{PML})\, \xi_{\mathrm{MD}} \cos\varphi]. \quad (60)$$

Here, $\xi_{\mathrm{MI(MD)}}$ corresponds to the spin current efficiency of magnetic-independent (magnetic-dependent) spinto-to-charge conversion and c is a coefficient constant. Both measurements under +M_{PML} and -M_{PML} can be well fitted by supplementary eq. (60) with $\xi_{\mathrm{MI}} > 0$ and $\xi_{\mathrm{MD}} < 0$ which is consistent with the damping-modulation measurement shown as figure 4b in the main text.

Moreover, we conducted the experiment in other samples with various Ni concentration and evaluated the absolute value ratio of conversion efficiency ξ_{MD} to ξ_{MI} (defined as $|\xi_{MD}/\xi_{MI}|$) shown in supplementary figure 10(d). The ratio shows monotonic increase with Ni concentration indicating that ξ_{MD} is sensitive to the interface structure. The Ni concentration dependence of the $|\xi_{MD}/\xi_{MI}|$ show consistent result with the linewidth modulation experiment (open star symbol) which is obtained from figure 4c in the main text.

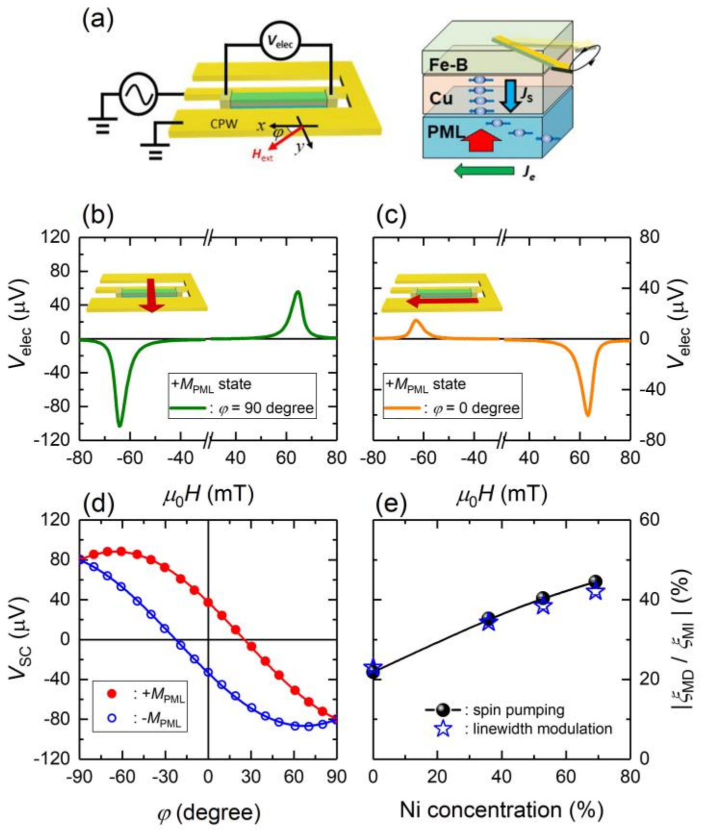

Supplementary figure 10: Spin-pumping induced spin-to-charge conversion in tri-layer structure. a Experimental set up for the spin pumping measurement (left) and schematic view of the spin-to-charge conversion in PML (right). Spin pumping induced electromotive force in $+M_{PML}$ state under **b** $\varphi = 90$ degree and **c** $\varphi = 0$ degree. Applied frequency and rf power of the coplanar waveguide (CPW) is set to be 3 GHz and 15 dBm, respectively. **d** In-plane angular dependence of V_{SC} under $+M_{PML}$ and $-M_{PML}$ state (solid red and open blue circles respectively). Solid line shows the fitting result. **e** Ni concentration dependence of the absolute value of $|\xi_{MD}/\xi_{MI}|$. Data obtained from spin pumping measurement (linewidth modulation measurement) is shown as black circles (blue open star symbols). Solid line is guide for eyes.

Supplementary Note 9:
SOT-induced field-free switching using in-plane magnetized ferromagnet source

We studied ξ_{MD} in perpendicularly magnetized ferromagnetic material which generates spin current with $\boldsymbol{x}$-axis spin polarization. If ξ_{MD} exists in ferromagnetic material, we can generate out-of-plane spin polarized spin current by changing the magnetization configuration of the spin source ferromagnetic material. To confirm this, we carried out additional experiments to check this through SOT-induced magnetization switching. To generate out-of-plane spin polarized (or z-axis spin polarized) spin current via ξ_{MD}, ferromagnetic spin source layer needs to be magnetized parallel (or antiparallel) to the applied charge current. For this purpose, we prepared a tri-layer system consisting of in-plane magnetized spin source layer (IML) and perpendicularly magnetized free layer (PML). As explained in the main text, Ni-Co/Cu interface show negative ξ_{MD}. Accordingly, expected spin polarization of ξ_{MD} under $\mathbf{M}_{IML}$ // +$\mathbf{x}$ ($\mathbf{M}_{IML}$ // -$\mathbf{x}$) in +$\mathbf{z}$ (-$\mathbf{z}$) direction when the charge current is applied to +$\mathbf{x}$ direction (Supplementary figure 11(a)). Here, we define the magnetization vector of IML as $\mathbf{M}_{IML}$. Therefore, the SOT-induced switching in PML is expected to show anti-clockwise (clockwise) chiral switching under $\mathbf{M}_{IML}$ // +$\mathbf{x}$ ($\mathbf{M}_{IML}$ // -$\mathbf{x}$) configuration.

To experimentally confirm the above consideration, we fabricated tri-layer based system: Ta-B (2.0) / Ru (2.0) / IrMn (6.0) / $Co_{70}Fe_{30}$ (1.5) / $Ni_{35}Co_{65}$ (2.5) / Cu (3.0) / Tb-Fe-Co (2.3) / MgO / Ta (2.0) (thickness in nm). The top Ta layer is fully oxidized during h the device fabrication process. Here, $Co_{70}Fe_{30}$ / $Ni_{35}Co_{65}$ bilayer corresponds to IML and Tb-Fe-Co ferrimagnet layer corresponds to PML. IrMn layer is deposited underneath the IML to pin $\mathbf{M}_{IML}$. As mentioned in the supplementary Note 2, we confirmed negligible interlayer exchange coupling in 3-nm thick Cu spacer, and thus the interlayer exchange coupling effect [22] does not affect the switching behavior in the present system. The sample is annealed at 250ºC for an hour in vacuum under 1T in-plane magnetic field. Instead of Co/Ni multilayer, we used $Co_{70}Fe_{30}$ in the present system to obtain strongly pinned IML exchange biased from IrMn under thermal annealing process. Supplementary figure 11(b) and 11(c) show the hysteresis curve of magnetic moment under magnetic field along $\boldsymbol{x}$ and $\boldsymbol{z}$ axis (H_x and H_z). From the in-plane field shift of ~100mT, we confirmed that the exchange bias of IrMn layer pins the IML layer along x-axis resulting in a uniform magnetization of IML at zero field. In addition, square hysteresis curve under out-of-plane field sweep shows an existence of PML free layer.

The film is fabricated into 20 μm-wide Hall bar structure and magnetization of PML is detected through anomalous Hall resistance R_{xy}. SOT-induced magnetization switching was observed by applying current pulse of 100 μs. Supplementary figure 11(d) and 11(e) show SOT-induced magnetization switching under $\mathbf{M}_{IML}$ // +$\mathbf{x}$ and $\mathbf{M}_{IML}$ // -$\mathbf{x}$ configuration in absence of external field.

The switching chirality shows opposite sign with reversing the magnetization of IML. More than three samples were tested, and we confirmed that the same tendency was reproduced in all the sample. The sign of the switching chirality is well explained by the ξ_{MD} mentioned above. We note that the present switching behavior corresponds to switching of 20~25% of magnetic domains, compared with the field driven R_{xy} loop. This partial switching was observed even when the in-plane assist field ±10mT is applied. The partial switching may originate from the demagnetization process from the current-induced heating effect in PML which was also mentioned in other works [23]. Although no full switching was observed, the chiral switching behavior with strong $\mathbf{M}_{\mathrm{IML}}$ dependence evidences the ξ_{MD} in our system and its contribution to the current-induced magnetization dynamics.

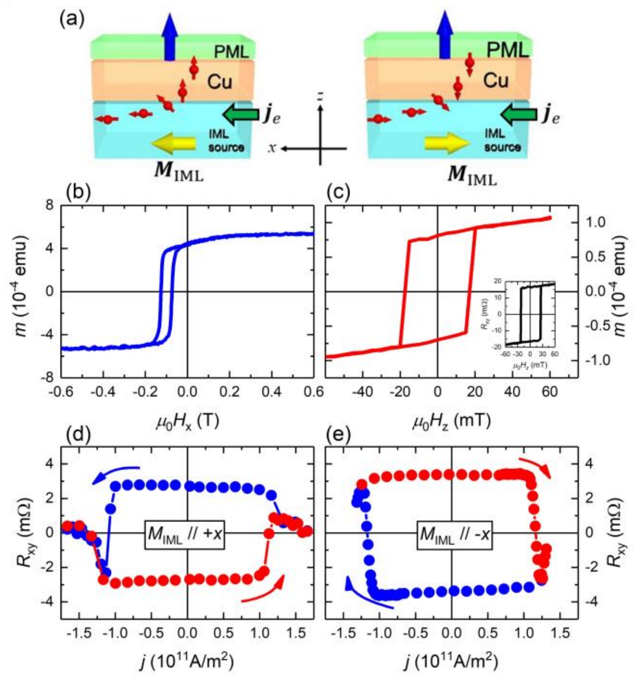

Supplementary Figure 11: Demonstration of SOT-induced field-free switching. a Schematic view of the IML/Cu/PML tri-layer structure and the charge-to-spin conversion in IML/Cu interface. Left (right) panel shows the spin current generation of +**z** (-**z**) polarized spin current with IML magnetized along +**x** (-**x** direction) when the charge current is applied along +**x** direction. **b** and **c** Magnetic moment under **x**-axis in-plane field **b** and **z**-axis out-of-plane field **c**. Both figures show the magnetization curve with M_{IML} // +**x** configuration. Inset shows R_{xy} under out-of-plane field. (**d** and **e**) SOT-induced magnetization switching in absence of external field under **d** M_{IML} // +**x** and **e** M_{IML} // -**x** configuration.

Supplementary References:

[1] M. Konoto, and S. Yuasa, Effect of MgO Cap Layer on Gilbert Damping Constant of FeB Electrode Layer in MgO-based Mangetic Tunnel Junctions, Appl. Phys. Express **6**, 073002 (2013).

[2] S. Mizukami, Y. Ando, and T. Miyazaki, Effect of spin diffusion on Gilbert damping for a very thin permalloy layer in Cu/Permalloy/Cu/Pt films, Phys. Rev. B **55**, 104413 (2002).

[3] Y. Shiota, S. Murakami, F. Bonell, T. Nozaki, T. Shinjo, and Y. Suzuki, Quantitative Evaluation of Voltage-Induced Magnetic Anisotropy Change by Magnetoresistance Measurement, Appl. Phys. Express **4**, 043005 (2011).

[4] A. Capua, S. H. Yang, T. Phung, and S. S. P. Parkin, Determination of intrinsic damping of perpendicularly magnetized ultrathin films from time-resolved precessional magnetization measurements, Phys. Rev. B 92, 224402 (2015).

[5] T. Taniguchi, S. Yakata, H. Imamura, and Y. Ando, Determination of Penetration Depth of Transverse Spin Current in Ferromagnetic Metals by Spin Pumping, Appl. Phys. Express **1**, 031302 (2008).

[6] T. Taniguchi and H. Imamura, Spin Pumping in Ferromagnetic Multilayers, Mod. Phys. Lett. B **22**, 2909 (2008).

[7] S. Zhang. P. M. Levy, and A. Fert, Mechanisms of Spin-Polarized Current-Driven Magnetization Switching, Phys. Rev. Lett. **88**, 236601 (2002).

[8] A. Brataas, Y. V. Nazarov, and G. E. W. Bauer, Spin-transport in multi-terminal normal metal-ferromagnet systems with non-collinear magnetizations, Eur. Phys. J. B **22**, 99 (2001).

[9] K.-W. Kim and K.-J. Lee, Generalized Spin Drift-Diffusion Formalism in the Presence of Spin-Orbit Interaction of Ferromagnets, Phys. Rev. Lett. **125**, 207205 (2020).

[10] V. P. Amin, J. Li, M. D. Stiles, and P. M. Haney, Intrinsic spin currents in ferromagnets, Phys. Rev. B **99**, 220405 (2019).

[11] G. Qu, K. Nakamura, and M. Hayashi, Magnetization direction dependent spin Hall effect in *3d* ferromagnets, Phys. Rev. B **102**, 144440 (2020).

[12] T. Valet and A. Fert, Theory of the perpendicular magnetoresistance in magnetic multilyaers, Phys. Rev. B **48**, 7099 (1993).

[13] T. Taniguchi, J. Grollier, M. D. Stiles, Spin-Transfer Torques Generated by the Anomalous Hall Effect and Anisotropic Magnetoresistance, Phys. Rev. Applied **3**, 044001 (2015).

[14] V. P. Amin and M. D. Stiles, Spin transport at interfaces with spin-orbit coupling: Phenomenology, Phys. Rev. B **94**, 104419 (2016), *ibid* 104420 (2016).

[15] V. P. Amin, J. Zemen, and M. D. Stiles, Interface-generated spin currents, Phys. Rev. Lett. **121**, 136805 (2018).

[16] G. G. B. Flores, A. A. Kovalev, M. van Schilfgaarde, and K. D. Belashchenko, Generalized

magnetoelectronic circuit theory and spin relaxation at interfaces in magnetic multilayers, Phys. Rev. B **101**, 224405 (2020).

[17] Y. Tserkovnyak, A. Brataas, and G. E. W. Bauer, Enhanced Gilbert Damping in Thin Ferromagnetic Films, Phys. Rev. Lett. **88**, 117601 (2002).

[18] K. Yakushiji, T. Saruya, H. Kubota, A. Fukushima, T. Nagahama, S. Yuasa, and K. Ando, Ultrathin Co/Pt and Co/Pd superlattice films for MgO-based perpendicular magnetic tunnel junctions, Appl. Phys. Lett. 97, 232508 (2014).

[19] K. Ando, S. Takahashi, J. Ieda, Y. Kajiwara, H. Nakayama, T. Yoshino, K. Harii, Y. Fujikawa, M. Matsuo, S. Maekawa, and E. Saitoh, Inverse spin-Hall effect induced by spin pumping in metallic system, J. Appl. Phys. **109**, 103913 (2011).

[20] L. Chen, M. Decker, M. Kronseder, R. Islinger, M. Gmitra, D. Schuh, D. Bougeard, J. Fabian, D. Weiss, and C. H. Back, Robust spin-orbit torque and spin-galvanic effect at the Fe/GaAs (001) interface at room temperature, Nat. Commun. **7**, 13802 (2016).

[21] Y. Shiomi, K. Nomura, Y. Kajiwara, K. Eto, M. Novak, K. Segawa, Y. Ando, and E. Saitoh, Spin-Electricity conversion Induced by Spin Injection into Topological Insulators, Phys. Rev. Lett. **113**, 196601 (2014).

[22] Y. C. Lau, D. Betto, K. Rode, J. M. D. Coey, and P. Stamenov, Spin-orbit torque switching without an external field using interlayer exchange coupling, Nat. Nanotehcnol. **11**, 758 (2016).

[23] H. Wu, Y. Xu, P. Deng, Q. Pan, S. A. Razavi, K. Wong, L. Hunag, B. Dai, Q. Shao, G. Yu, X. Han, J.-C. Rojas-Sánchez, S. Mangin, and K. L. Wang, Spin-Orbit Torque Switching of a Nearly Compensated Ferrimagnet by Topological Surface States, Adv. Mater. **31**, 1901681 (2019).
