# Supplementary material for: Giant charge-to-spin conversion in ferromagnet via spin-orbit coupling
Source: Nat Commun. 2021 Oct 29;12:6254. doi: 10.1038/s41467-021-26445-y (PMC8556288; doi:10.1038/s41467-021-26445-y)
Supplement: Supplementary file 3 — Editorial Assessment Report [file 41467_2021_26445_MOESM3_ESM.pdf]

nature portfolio

GUIDED OPEN ACCESS

nature physics | nature communications | communications physics

Editorial Assessment Report

Dear Dr Yuki Hibino,

Thank you again for choosing to submit your manuscript using the Guided Open Access pilot at the Nature Portfolio. As part of this process, our editorial team has considered your paper for three of our journals with strong interest in publishing in your field: *Nature Physics*, *Nature Communications*, and *Communications Physics*.

Your manuscript has now been reviewed by three experts in spintronic devices and magnetisation switching from both and experimental and theoretical perspective. We have included their comments in this Editorial Assessment Report. As part of the Guided Open Access pilot, editors from all three journals have discussed the reviewer reports and the manuscript's suitability for our journals. After careful evaluation, our editorial recommendation is to revise the manuscript and submit back through the Guided Open Access submission portal for consideration at *Communications Physics*. Provided the revisions satisfy all technical and editorial concerns, *Communications Physics* is interested in publishing your manuscript. Please see details in the attached Editorial Assessment Report.

In brief, for publication in Communications Physics, we would require you to address the referee concerns regarding the discussion of the underlying mechanisms as best you can, as well as addressing all other technical concerns raised. You will find more details regarding the required revisions in the Editorial Assessment Report below.

Please note that the Editorial Assessment Report is a standalone document that contains an editorial evaluation, recommendation and portable peer advice to help you navigate and interpret the reviewers' reports. It also provides guidance for adhering to best practice with regard to transparency and reproducibility, for example on the issue of sharing data. We have also included information about data accessibility and reproducibility, which we hope you find useful.

Yours sincerely,

Dr Saleem Denholme
Senior Editor, *Communications Physics*
On behalf of the Guided OA editorial team

Contents of this Report

- **Manuscript overview**: details about your manuscript and the editorial team.
- **Manuscript assessment**: personalised recommendation from the editors.
- **About the editorial process**: an overview of the Guided Open Access process.
- **Annotated reviewer comments**: the referee reports with comments from the editors.
- **Open research evaluation**: advice for adhering to best reproducibility practices.

Manuscript overview

Manuscript number	Submission date	Decision date
GUIDEDOA-21-00047	21 February 2021	Click or tap to enter a date.

**Title**	Giant charge-to-spin conversion in ferromagnet via spin-orbit coupling	**Corresponding author**	Yuki Hibino<br>ORCID: 0000-0002-2297-1786
**Preprint information**	None found	**Peer review type**	Single-blind
**Editorial Assessment Team**	**Primary editor: Saleem Denholme**<br>**Home Journal:** *Communications Physics*, ORCID: 0000-0001-6017-0819<br>**Editorial team members:**<br>Samuel Bladwell, *Nature Communications*, ORCID: 0000-0002-2805-3206<br>David Abergel, *Nature Physics*, ORCID: 0000-0002-6166-181X		
**About your primary editor**	Saleem joined Communications Physics in October 2017. He obtained his PhD from the University of Glasgow investigating the synthesis and characterisation of transition metal chalcogenide nanomaterials. He then conducted postdoctoral work as a Japanese Society for the Promotion of Science Fellow at the National Institute of Materials Science, Tsukuba and later as assistant professor in the Department of Applied Physics at the Tokyo University of Science. His research interests include low-dimensional materials and iron-based superconductivity. At Communications Physics Saleem handles manuscripts in materials science and condensed matter physics. Saleem is based in the London office.		

Manuscript assessment and recommendation

**Editor's summary of the manuscript and overall assessment**	The authors report an enhanced charge to spin conversion in a ferromagnetic and normal metal heterostructure. Using ST-FMR measurements they clarify the mechanisms for the bulk and surface contribution to the spin torque as a function of layer thickness and chemical composition. They propose a spin-Hall precession effect as novel parameter of the bulk component.

Editorial assessment overview

Nature Physics	**Not feasible to meet the editorial requirements**	Does not provide enough advance in terms of performance or novel insight into the mechanisms of magnetisation switching for *Nature Physics*.
Nature Communications	**Major revisions needed**	Currently unsuitable, but may reconsider if more methods can be used to explore a deeper understanding of the results.
Communications Physics	**Minor revisions needed**	Will consider for publication in *Communications Physics* once all reviewer concerns have been addressed.

**Editor's recommendation**	***Option 1*: Revise for consideration at Communications Physics**<br>While revision is needed, the majority of concerns raised should be resolvable. The most pertinent of which are those regarding the underlying mechanisms behind the enhanced charge to spin conversion. See the reviewer comments for details.<br><br>***Option 2*: Revise for consideration at Nature Communications**<br>If a deeper understanding of the results can be provided by performing additional experiments or analysis including but not limited to the suggestions from the reviewers, the manuscript may be reconsidered for publication for publication in Nature Communications.

**Next steps**	If you would like to follow our recommendation, when you are ready you can upload the revised manuscript, along with your point-by-point response to the reviewer's reports and editorial advice **here**.

About the editorial process

By selecting the Nature Portfolio Guided Open Access option, your manuscript was assessed for suitability in three of our titles that provide venues for publication of high-quality work across the spectrum of physics research: *Nature Physics, Nature Communications*, and *Communications Physics*. For more information about Guided Open Access, please see here.

Collaborative editorial assessment

Your editorial team discussed the manuscript to determine its suitability for the Nature Portfolio Guided OA pilot. Our assessment of your manuscript takes into account several factors, including whether the work meets the **technical standard** of the Nature Portfolio and whether the findings are of **immediate significance** to the readership of at least one of the participating journals in the Nature Portfolio Guided Open Access physics cluster.

Peer review

Experts were asked to evaluate the following aspects of your manuscript:

Novelty in comparison to prior publications;

Likely audience of researchers in terms of broad fields of study and size;

Potential impact of the study on the immediate or wider research field;

Evidence for the claims and whether additional experiments or analyses could feasibly strengthen the evidence;

Methodological detail and whether the manuscript is reproducible as written;

Appropriateness of the literature review.

Editorial evaluation of reviews

Your editorial team discussed the potential suitability of your manuscript for each of the participating journals. They then discussed the revisions necessary in order for the work to be published, keeping each journal's specific editorial criteria in mind.

Journals in the Nature portfolio will support authors wishing to transfer their reviews and (where reviewers agree) the reviewers' identities to journals outside of Springer Nature. For any questions about review portability, please contact our editorial office: guidedoa@nature.com

Annotated Reviewer Reports

The editor has included some additional comments on the specific points raised by the reviewers below. However, please note that all points should be addressed in a revision, even if the editor has not specifically commented on them.

Reviewer #1

Report received: 10 April 2021

**Reviewer #1**	This reviewer has not chosen to waive anonymity. The reviewer's identity can only be shared with representatives of an established journal editorial office.
**Reviewer #1 expertise** *Summarised by the editor*	This reviewer has expertise in experimental spintronics, spin orbit and transfer torques.
**Editor's comments about this review**	The reviewer believes the results are of interest to those studying methods to enhance SOT efficiency and field-free switching using FM layers. They outline some minor technical concerns/comments which should be straightforward for the authors to address.

Reviewer #1 comments

**Overview**	The authors have used ST-FMR method to find out the origin of spin torque rising from FM/NM structure. This is a quite interesting research and provide some insight that may enhance SOT efficiency and possibly field-free switching with FM layers. I think the results are quite reasonable and well organized.

Specific comments

#	Reviewer comment	Editorial comment
**Technical comments**		
**1**	I suppose the main reason for Fig.2(c) is to show that the slope of –MPML and +MPML are different, which is one of reasons that supports the existence of magnetization dependent torque. Then, I think the authors need to find out a good way to show that the slopes of blue and red lines are different. In current configuration, it is difficult to know if there are any differences between the two lines.	It is essential to carefully revise the presentation used in the manuscript to avoid any confusion with the main results.
**2**	In Fig.3(a), why are Series A and B fitted with a same line?	

	Shouldn't there be separate fitting lines for Series A and B?	
3	With Co/Ni or Co/Pt, the authors showed that the sign of spin-orbit precession and the spin hall precession effect compete with each other. Any suggestions on how to reverse the sign of the spin-orbit precession effect or the spin Hall effect in FM in order to enhance the efficiency?	
**Reproducibility**		
4	I think a concrete evidence of spin hall precession effect would be to show a difference in spin torque effect between –MPML and +MPML. Can the authors provide this evidence? Or do the authors think Figure 2(a) supports this phenomenon?	Providing further evidence for the spin Hall precession effect will increase the strength of your results. Please make sure to address this comment as best you can.
5	The authors did 'some' experiments to verify the origin of the torques rising from FM layer. Basically current flowing in x-direction in FM materials with z-magnetization can provide both y-direction spin orientation (MI) and x-direction spin orientation (MD). I think this is an interesting paper, that gives some new approach to field free switching and highly efficient SOT devices.<br><br>Unfortunately, the experiment only considers ST-FMR as the evaluation method. If there were another method like current induced SOT switching to cross-check their experimental data, then the paper may be suitable for journals like Nature Communications (It does not necessarily have to be SOT switching, just another different method to cross-check). But in current situation, I think Communications Physics is the suitable journal for publication, if the authors can give good answers to my comments.	For Communications Physics it is optional whether you wish to address this comment or not.<br>For a chance at Nature Communications, more methods to evaluate the results are needed in hope to gain a deeper understanding.

**Reviewer #2**<br>*Report received:* 14 April 2021	
**Reviewer #2**	This reviewer has not chosen to waive anonymity. The reviewer's identity can only be shared with representatives of an established journal editorial office.

**Reviewer #2 expertise** *Summarised by the editor*	This reviewer has expertise in the theory of spintronics for a range of materials
**Editor's comments about this review**	The reviewer finds the study provides a useful insight into the various spin current generation mechanisms in ferromagnetic trilayers. They request further clarification on the bulk mechanism and for the authors to consider mechanistic explanations other what has already been proposed. The referee would also like more details on the motivation behind the setup used and more information on the interface quality of each sample.
**Reviewer #2 comments**	
**Overview**	The authors perform ST-FMR measurements in ferromagnetic trilayers to probe unconventional spin current generation, both from the fixed ferromagnetic layer and from the interface between that layer and the nonmagnetic spacer layer. By varying the thickness of the fixed layer (with perpendicular magnetization) and the Co-Ni concentration near the interface, the strength of the bulk and interfacial contributions to the torques are studied. The unconventional torques are an order of magnitude larger than those reported in other systems that break the required mirror symmetries, such as transition-metal dichalcogenides and non-collinear antiferromagnets. The authors then propose a phenomenological mechanism by which spin Hall currents rotate their spin direction about the magnetization in ferromagnets to explain the bulk-originating contribution to the unconventional (m x j x z) torques. The manuscript is fairly well written, and the results are clearly described in the main text and figures. One of the main contributions beyond previous work is the study of interfacial composition on the unconventional torques and (to a lesser extent) the proposed theoretical mechanism. Given a few revisions, I believe this work warrants publication in one of the suggested Nature journals. I have a few comments that I list below:

Specific comments

#	Reviewer comment	Editorial comment
**Technical Comments**		
1	The mechanism suggested for bulk spin current generation in ferromagnets, called the "spin Hall precession effect" by the authors, is supported by drift-diffusion calculations but not the only plausible mechanism. It is simpler and more accurate to say that the bulk-originating spin current is allowed by symmetry in ferromagnets and could arise from multiple mechanisms, since it is unclear which mechanism	The editor strongly recommends you provide a more thorough discussion of the underlying mechanisms as suggested by the referee. This is likely to widen the insight which your study can provide as well as potentially trigger follow up work by the

	dominates. Alternate mechanisms have already been suggested by other researchers (aside from spin swapping, which is mentioned in the manuscript). For instance, in Phys. Rev. Lett. 121, 136805 (which the authors already cite regarding interface-generated spin currents), a bulk mechanism is introduced that generates "spin-orbit precession-like" currents in infinite Co leads, supported by first principles transport calculations. The authors state that the origin of this effect is related to the planar Hall effect. Furthermore, various authors have reported on the so-called "magnetic spin Hall effect" (Phys. Rev. Research 2, 023065) which allows for spin currents of the same orientation as discussed above. Note that the spin currents discussed in both references have a similar underlying origin. Thus, given that there are several pathways for bulk spin currents to exist with spin direction along m x (j x z) (with some higher-order contributions), it would seem best to refrain from singling out a particular bulk mechanism. The authors could certainly suggest that the "spin Hall precession effect" is a candidate but should inform the readers of other possibilities.	community.
**2**	The motivation of this paper is the control of out-of-plane magnetizations, yet in this work the source layer has an out-of-plane magnetization and the free layer has an in-plane magnetization. The authors should at least comment on why this orientation was chosen. There are two cases of interest: 1) z-magnetization, x-electric field, z-spin flow, x-spin direction and 2) x-magnetization, x-electric field, z-spin flow, z-spin direction. Both cases are allowed by symmetry in ferromagnets. The first case is studied in this experiment whereas the latter is more useful for controlling magnetization dynamics in PML films. Certainly, both cases are related by the parameter σ_MD, but given higher-order-in-magnetization contributions, the spin currents in each of these cases could be different. It would be useful for the authors to at least discuss how the measurements done in this geometry will help optimize the control of out-of-plane magnetizations in other geometries.	Again, the editor strongly recommends you follow this advice which is likely to increase the usefulness of the results to the community.
**Comments on impact**		
**3**	I believe this paper provides useful insight into the various spin current generation mechanisms in ferromagnetic	

	trilayers.	
**Reproducibility**		
4	Overall, I consider the results and conclusions to be fairly convincing. However, it is worth raising an additional concern. Thickness-dependent measurements are one of the few ways to isolate interfacial contributions. However, since they require measurements to be performed over different samples, it is difficult to ensure that each sample has a similar interface, and that only the thickness of one layer is significantly different. However, given the lack of better approaches, it is worth performing these experiments. The results could be strengthened if more was said about the quality of the interfaces in each of the samples.	

**Reviewer #3**<br>*Report received:* 12 April 2021	
**Reviewer #3**	This reviewer has not chosen to waive anonymity. The reviewer's identity can only be shared with representatives of an established journal editorial office.
**Reviewer #3 expertise** *Summarised by the editor*	The reviewer has expertise in AFM-spintronics and control of magnetisation
**Editor's comments about this review**	The reviewer believes the reported giant charge-to-spin conversion efficiency is an exciting result but more must be done to clarify the underlying mechanisms.
**Reviewer #3 comments**	
**Overview**	In this manuscript, the authors demonstrate strong charge-to-spin conversion in ferromagnet. They utilize Co/Ni multilayers as the spin source, which shows bulk perpendicular magnetic anisotropy and benefits the thickness dependent analysis. This configuration also excludes the spin anomalous Hall effect as the origin. Both theoretical calculation and experimental results are concise, and the giant charge-to-spin conversion efficiency, which is an order of magnitude larger than the

	materials with reduced symmetry such as WTe2, is exciting. However, the mechanism is still not clarified clearly enough as following, therefore I suggest this work to be further evaluated by *Communication Physics*.

Specific comments

#	Reviewer comment	Editorial comment
**General comments**		
**1**	In abstract, it is declared that "The interfacial contribution to charge-to-spin conversion is found to be dominant". However, this is only true when the Co/Ni multilayer is thin. The bulk contribution is also non-negligible from the thickness dependence result.	
**Comments on mechanism**		
**2**	The detailed mechanism of bulk spin-Hall precession is not explained clearly. Why such spin would not undergo dephase? Why it would precess from one orthogonal-to-magnetization direction to the other?	The editor strongly advises that any ambiguities in the underlying mechanism are clarified. This will increase the impact and appeal of your study.
**3**	The spin-orbit precession is still dominant for ξMD in this system. I don't understand why they could realize such a large efficiency. Besides, this comparison is based on the materials with reduced symmetry, but the mechanisms are totally different. What if compare to the similar ferromagnetic trilayers.	
**4**	In Fig. 3a, magnetic dependent spin current conductivity decreases with the increase of the PML thickness. But the theoretical fitting shows an opposite tendency when the PML thickness is very small. How do you explain this divergency between calculating and experiment? Since the interface and bulk have opposite contribution to the charge-to-spin conversion, is it possible to manipulate the sign of magnetic dependent spin current conductivity by further increase the PML layer thickness?	
**5**	The authors claim that both the Rashba effect and the spin polarization contribute to the interface-related charge-to-spin conversion, but they did not explain why. If these two effects are linear with the Ni concentration and have opposite sign, why ξMD drop at Ni 100%?	

Other		
6	The authors should clarify that the solid lines in Fig. 4c are just guide to the eye.	

Open Research Evaluation	
**Data Availability**	
**Recommended data deposition**	The Nature Portfolio journals strongly support public availability of data associated with a manuscript in a persistent repository where they can be freely and enduringly accessed or as a supplementary data file when no appropriate repository is available. For more information, please refer to our page on reporting standards and availability of data, materials, code and protocols.<br><br>We recommend that all relevant raw or processed experimental data in your study be made available at the point of publication on a public repository such as Figshare or Zenodo, so that they can be referred with unique digital object identifiers.<br><br>In addition, we strongly encourage you to upload the Source Data (that is, the specific data shown in the figures in the main text and Extended Data) along with your resubmission.<br><br>If you need help complying with this policy, or need help depositing and curating your research data (including raw and processed data, text, video, audio and images) you should consider:<br>• Contacting Springer Nature's Research Data Helpdesk for advice,<br>• finding a suitable data repository for your data,<br>• Or uploading your data to Springer Nature's Research Data Support service.<br><br>Please note there are fees for using Springer Nature's Research Data Support service. You may also find more information on our policy page.
**Data Availability Statement**	All papers published by the Nature Portfolio must include a Data Availability Statement (DAS).<br><br>A DAS should include, at a minimum, a statement confirming that all relevant data are available from the authors, and/or are included with the manuscript (e.g. as source data or

	supplementary information), listing which data are included (e.g. by figure panels and data types) and mentioning any restrictions on availability. If a dataset generated or analysed during the study is publicly available and has a Digital Object Identifier (DOI) as its unique identifier, we strongly encourage including this in the Reference list and citing the dataset in the Methods.<br><br>In the present case, we suggest that you amend your DAS as follows:<br><br>“Source data are available for this study. All other data that support the plots within this paper and other findings of this study are available from the corresponding author upon reasonable request”.<br><br>However, please feel free to amend this statement, especially if you are able to share more of your data.
**Source data**	The following figure panels should be accompanied by the underlying source data: **Figs 2 and 3**.
**Data citation**	If a dataset generated or analysed during the study is publicly available and has a DOI as its unique identifier, we strongly encourage including this in the Reference list and citing the dataset in the Methods.<br><br>Citing and referencing data in publications supports reproducible research, by increasing the transparency and provenance tracking of data generated or analysed during research. Citing data formally in reference lists also helps facilitate the tracking of data reuse and may help assign credit for individuals’ contributions to research. A number of Springer Nature imprints are signatories of the Joint Declaration on Data Citation Principles, which stress the importance of data resources in scientific communication.
**Code Availability**	
**Code Availability**	As for data, the Nature Portfolio journals strongly support public availability of custom code associated with a manuscript in a persistent repository where they can be freely and enduringly accessed, where appropriate.

**Code Citation**	In addition to making the custom code available, we ask that you ensure that the version of the code/software described in the paper is deposited in a DOI-minting repository such as Zenodo and that this DOI is also cited in the main Reference list. See here for more details.
**Research ethics**	
**Competing Interests**	In the interests of transparency and to help readers form their own judgements of potential bias, Nature Portfolio journals require authors to declare any competing financial and/or non-financial interests in relation to the work described.<br>Please provide a 'Competing interests' statement using one of the following standard sentences:<br>• The authors declare the following competing interests: [specify competing interests]<br>• The authors declare no competing interests.<br>See our competing interests policy for further information: https://www.nature.com/nature-research/editorial-policies/competing-interests
**Methods assessment and reproducibility**	
**Methods Descriptions**	The Methods must contain sufficient detail such that the work could be replicated. It is preferable that all key methods be included in the main manuscript, rather than in the Supplementary Information.<br>The methods section can be up to 3,000 words in length, they can contain references that do not count towards the reference limit in the main paper, and will be fully indexed. You should feel free, and we in fact encourage you, to incorporate any part of your Supplementary Information that you feel is important for the rest of the paper within this section.<br>The Methods section should be written as concisely as possible but should contain all elements necessary to allow interpretation and reproduction of the results (please note, however, that the methods section cannot contain any

	figures or tables at present).<br>If there are additional references in the Methods section, their numbering should continue from the last reference in the main paper, and the list should follow the Methods section.
**Error bars and statistics**	Error bars should be displayed wherever possible and must be clearly defined in the caption for each figure.
